# Outpatient Versus Inpatient Administration of Ciltacabtagene Autoleucel in Multiple Myeloma: A Systematic Review of Clinical, Economic, and Humanistic Outcomes

**DOI:** 10.3390/cancers18050755

**Published:** 2026-02-26

**Authors:** Tara Gregory, Kevin C. De Braganca, Victoria Alegria, Matthew Perciavalle, Ravi Potluri, Sandip Ranjan, Todd Bixby, Zaina P. Qureshi

**Affiliations:** 1 Sarah Cannon Cancer Network, Colorado Blood Cancer Institute, Denver, CO 80218, USA; tara.gregory@healthonecares.com; 2 Johnson & Johnson, Raritan, NJ 08869, USA; kdebraga@its.jnj.com; 3 Janssen Scientific Affairs, Horsham, PA 19044, USA; valegria@its.jnj.com (V.A.); tbixby@its.jnj.com (T.B.); 4 Legend Biotech USA, Inc., Somerset, NJ 08854, USA; matthew.perciavalle@legendbiotech.com; 5 Putnam Associates, New York, NY 10282, USA; ravi.potluri@putassoc.com; 6 Putnam Associates, Gurgaon 122002, India; sandip.ranjan@putassoc.com

**Keywords:** ciltacabtagene autoleucel, multiple myeloma, CAR-T, outpatient administration, effectiveness, safety, cost

## Abstract

**Background/Objectives**: Ciltacabtagene autoleucel (cilta-cel) for relapsed/refractory multiple myeloma is typically administered inpatient (IP) to monitor for cytokine release syndrome (CRS) and immune effector cell-associated neurotoxicity syndrome (ICANS). Because cilta-cel toxicities are typically delayed, outpatient (OP) administration (infusion and early monitoring) is being explored. We synthesized available evidence on OP and IP administration. **Methods**: MEDLINE, Embase, and Cochrane Library were searched from inception to 5 August 2025, supplemented by conference and gray literature searches. Eligible studies of adults with multiple myeloma receiving cilta-cel reported efficacy, safety, resource use, costs, and/or quality-of-life outcomes; findings were synthesized descriptively due to heterogeneity. **Results**: Seventy-four records (56 studies) were included; 90 patients received OP cilta-cel. OP clinical evidence (primarily three real-world studies) showed high response rates (ORR: 95%; median follow-up 4.6 months) and reported 1-year PFS and OS of 86% and 96%. In IP studies, median ORR was 91%, with median 1-year PFS 76% and median 1-year OS 85%. Any-grade CRS and ICANS occurred in 79–84% and 17–22% of OP patients (largely low grade); IP cohorts reported a median ICANS incidence of 17% (range 5–23%). Most OP patients were later hospitalized (86–93%), but stays were shorter (median 4–6.5 days) than in an IP cohort (median 19 days). Comparisons were unadjusted and may reflect selection differences. One modeling-based economic analysis estimated savings of ~$19,000 per OP-treated patient. **Conclusions**: OP cilta-cel appears feasible for selected patients and may reduce costs without compromising outcomes. Findings are descriptive and hypothesis-generating and prospective multicenter studies are needed to define long-term safety, durability, quality of life, and cost-effectiveness.

## Simple Summary

Ciltacabtagene autoleucel (cilta-cel) is a CAR-T treatment for relapsed/refractory multiple myeloma. It is often given in the hospital so clinicians can watch for side effects such as cytokine release syndrome and neurologic symptoms. Because these side effects with cilta-cel usually start several days after infusion, some centers are exploring giving it as an outpatient. In this systematic review, we gathered and summarized evidence on outpatient and inpatient cilta-cel administration, covering effectiveness, safety, healthcare resource use, costs, and quality-of-life outcomes. The findings suggest potential advantages of outpatient administration with respect to hospitalization, healthcare resource utilization and costs, while highlighting the need for further prospective and comparative studies to more comprehensively characterize clinical, economic, and patient-reported outcomes.

## 1. Introduction

Multiple myeloma (MM) is a malignant plasma cell disorder that accounts for around 10% of all hematologic malignancies [1]. Despite advances in treatment over the past decade and more with proteasome inhibitors (PIs), immunomodulatory drugs (IMiDs), anti-CD38 monoclonal antibodies, antibody–drug conjugates (ADCs), bispecific antibodies (BsAbs), MM remains a relapsing disease for many patients, requiring successive lines of therapy [2,3,4]. Frontline regimens typically achieve high initial response rates; however, disease relapse driven by clonal evolution and treatment resistance is common, underscoring the need for therapies capable of inducing deeper and more durable responses in relapsed and refractory disease [5]

CAR-T cell therapy has emerged as a transformative option for patients with relapsed or refractory multiple myeloma (RRMM) [5,6,7]. One such therapy is ciltacabtagene autoleucel (cilta-cel), which received its first approval in February 2022 for heavily pretreated patients with RRMM [8,9]. Following results from the phase III CARTITUDE-4 trial, it gained expanded approval in April 2024 for use after ≥1 prior line(s) of therapy in lenalidomide-refractory disease [10]. Cilta-cel is a B-cell maturation antigen (BCMA)-directed CAR-T therapy incorporating two BCMA-binding domains, enabling high-avidity engagement of malignant plasma cells and potent T-cell activation, expansion, and cytotoxic tumor cell killing [11]. Cilta-cel has shown unprecedented response rates, with many patients achieving deep and durable remissions where prior treatments had failed [5,7,8,9,10]. Clinical trials and real-world studies show that cilta-cel improves progression-free survival (PFS), overall survival (OS), and quality of life (QoL) compared with standard of care in patients with RRMM who have received at least one prior line of therapy [5,8,9,10,12,13].

Notwithstanding its efficacy, CAR-T therapy can be associated with acute toxicities, most notably cytokine release syndrome (CRS) and immune effector cell-associated neurotoxicity syndrome (ICANS) [14]. CRS is a systemic inflammatory response resulting from rapid immune activation after CAR-T infusion, while ICANS encompasses a spectrum of neurologic manifestations such as confusion, aphasia, and seizures, all of which can substantially affect patients’ QoL and functional independence [15]. These toxicities may require inpatient (IP) administration and intensive monitoring leading to prolonged hospitalization, high healthcare resource utilization (HCRU), and substantial logistical and financial burden for both institutions and patients [14,16,17]. In contrast, cilta-cel exhibits a relatively delayed and predictable CRS and ICANS profile, with a median onset of approximately 7 and 8 days post-infusion, respectively, which are later than those observed with other CAR-T therapies [8,11,13,14]. While this delayed toxicity profile requires structured OP monitoring and rapid access to IP care if needed, it also offers greater feasibility for administering cilta-cel in the outpatient (OP) setting, which can reduce hospitalization burden, optimize healthcare resource utilization (HCRU), lower costs, and may have indirect benefits for patients’ QoL [8,16,17,18]. OP CAR-T administration refers to infusion without planned overnight hospitalization. It requires intensive early monitoring (often daily), dedicated caregiver support, proximity to the treating center, and predefined criteria for hospital admission in case of CRS, ICANS, or other complications. [19,20].

Retrospective real-world cohort studies and expert consensus suggest that, with careful patient selection, structured monitoring protocols, and dedicated caregiver support, cilta-cel can be safely administered in the OP setting [16,18]. However, evidence describing outcomes across IP and OP delivery remains limited. Important questions persist regarding whether OP administration can be implemented safely and effectively in selected patients and how this approach influences patient-reported outcomes (PROs), healthcare resource use, and overall costs. Addressing these questions is essential as CAR-T therapies expand into earlier treatment lines and broader patient populations.

The objective of this systematic literature review (SLR) was to describe the collective evidence on outpatient and inpatient administration of cilta-cel, comparing efficacy, safety, healthcare resource utilization, costs, and available QoL outcomes, for patients with RRMM.

## 2. Methods

### 2.1. Study Design and Search Process

This SLR was conducted in accordance with the Preferred Reporting Items for Systematic Reviews and Meta-Analyses (PRISMA) checklist [21] and the Cochrane Handbook for Systematic Reviews of Interventions (version 6.3) [22]. This SLR was not registered in the PROSPERO database. A comprehensive search of MEDLINE, Embase, and the Cochrane Library was conducted on 5 August 2025. The search included all records from database inception up to that date without any time restrictions. Records were retrieved based on a predefined search strategy developed using a combination of controlled vocabulary (Medical Subject Headings [MeSH] in MEDLINE and Emtree terms in Embase) and free-text keywords related to multiple myeloma and ciltacabtagene autoleucel. Following Cochrane recommendations, the PICOS framework (Population, Intervention, Comparator, Outcomes, Study design) was used to develop the search strategy and ensure comprehensive coverage of relevant clinical, economic, and humanistic evidence. The electronic database search strategies employed for the searches are provided in Tables S1–S3.

To ensure complete coverage of emerging evidence, targeted searches were performed in key conference proceedings and gray literature sources. These included conference proceedings of the American Society of Clinical Oncology (ASCO), European Society for Medical Oncology (ESMO), American Society of Hematology (ASH), European Hematology Association (EHA), International Society for Pharmacoeconomics and Outcomes Research (ISPOR), International Myeloma Society (IMS), Society of Hematologic Oncology (SOHO) and Academy of Managed Care Pharmacy (AMCP). Additional sources such as Google Scholar and disease-specific organizational websites were screened to capture recent presentations, unpublished data and studies that might not have been indexed in databases. Reference lists of relevant systematic reviews were manually examined to identify additional eligible records. A full list of all bibliographic databases, clinical trial registries, and online platforms searched is provided in Table S12.

### 2.2. Eligibility Criteria

Selection of relevant studies was conducted using predefined eligibility criteria structured according to the PICOS framework. Studies were included if they reported outcomes of cilta-cel in adult patients with MM. Outcomes of interest included efficacy, safety, health-related quality of life (HRQoL), HCRU, and costs. Any study design, including interventional clinical trials (phase I–III), observational studies (prospective or retrospective), economic models and clinician or administrative surveys related to OP use of cilta-cel were eligible for inclusion in the review. Multiple study designs were included to comprehensively capture clinical, economic, and operational evidence relevant to outpatient and inpatient administration, with findings synthesized descriptively while accounting for heterogeneity across study types. No restriction on geography was applied.

Non-human studies, reviews, editorials, commentaries, and studies not published in English were excluded. The details of the eligibility criteria are specified in Table S4.

**Figure 1:**
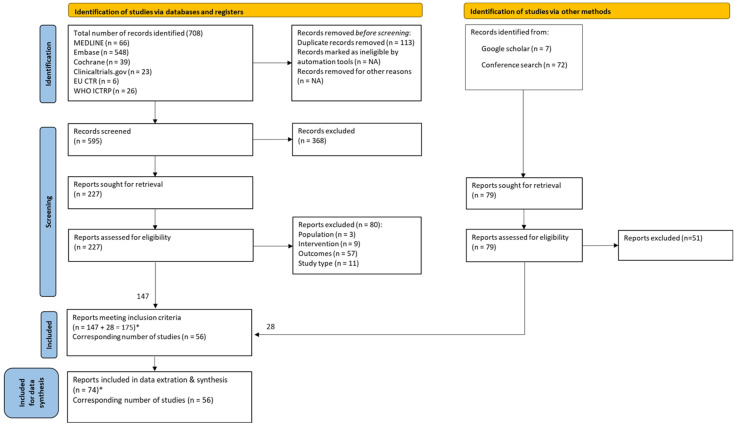
The Preferred Reporting Items for Systematic Reviews and Meta-Analysis (PRISMA) flow diagram. * Some studies had multiple reports; reports that were unique and had the most up-to-date outcomes were included for data synthesis.

A two-stage screening process was conducted using PICOS-based criteria and reported according to PRISMA 2020 guidelines. In the first stage, all retrieved citations were independently screened by two reviewers based on the title and abstract. Full texts for all records included in the first stage were retrieved for review in the second stage. In the second stage, citations were independently examined in full text by two reviewers to assess compliance with the eligibility criteria. Any differences were resolved through discussion, with arbitration by a third reviewer when necessary. Multiple reports of the same study were consolidated, and the unique records and most up-to-date outcomes were considered for data extraction and synthesis. The results of the screening process are presented in the PRISMA flow diagram (Figure 1).

### 2.3. Data Extraction

Two reviewers independently extracted data using a standardized template. Extracted items included study design, country, treatment setting (OP, IP, both or not reported), sample size, patient and disease characteristics, cilta-cel treatment details, and all outcomes of interest. Outcomes included efficacy (CR, PR, ORR, PFS, OS), safety (CRS, ICANS, other adverse events), HRQoL, HCRU (hospitalization rates, time to admission, length of stay, intensive care unit (ICU) admissions, OP visits), and costs. Any differences in extraction were discussed and resolved by consensus or with the involvement of a third reviewer.

### 2.4. Data Analysis

All extracted data were synthesized descriptively due to heterogeneity across study designs and outcomes; therefore, no statistical pooling or meta-analysis was performed. Study and patient characteristics, treatment setting, and outcomes were tabulated and summarized. Where possible, findings were stratified by administration setting (OP vs. IP). When studies reported combined OP and IP data only or where setting was not reported, results are presented for the combined cohort. Outcomes were reported separately for OP and IP cohorts; evidence from comparative and single-cohort studies was included. A formal assessment of methodological quality or risk of bias was not performed, as the review aimed to provide a descriptive synthesis across a highly heterogeneous evidence base spanning multiple study designs. Accordingly, findings were interpreted qualitatively, with consideration of differences in study design, follow-up duration, patient selection, and outcome reporting. Results are presented in summary tables and figures, with additional details available in the Supplementary Materials.

## 3. Results

Overall, evidence on OP administration of cilta-cel was limited and largely non-comparative. Given substantial heterogeneity across study designs, populations, and reported outcomes, findings were synthesized descriptively, and no statistical pooling or meta-analysis was performed. Across available studies, efficacy, safety, and healthcare resource utilization outcomes reported in OP cohorts generally fell within the ranges observed for IP settings. Although hospitalization after OP infusion was common, these admissions were often protocol-driven for monitoring or toxicity management and were typically short when length of stay was reported. Economic evaluations suggested potential cost advantages associated with OP administration, although findings were context-specific. Evidence on quality-of-life outcomes remains an important gap, as no OP-specific QoL data were identified. Overall, the results underscore the need for prospective, multicenter comparative studies to better characterize long-term safety, durability of response, QoL outcomes, and cost-effectiveness of OP cilta-cel administration.

### 3.1. Literature Search Results

Database searches identified 708 records. After deduplication, 595 records were screened, of which 147 met the eligibility criteria. An additional 28 records were identified from supplementary sources, resulting in 175 full-text records being included. Of these, 74 unique records reporting outcomes of cilta-cel were included in the data extraction and synthesis. The remaining 101 linked publications met the inclusion criteria but were not included in data extraction or synthesis as they represented dated or overlapping reports of the included studies (Figure 1).

### 3.2. Study Characteristics

The 74 records included for the data extraction corresponded to 56 unique studies (5 clinical trials [9 sub-studies], 34 retrospective observational studies, 2 prospective observational studies, 9 economic studies, 1 physician survey and 1 case series), with sample sizes ranging from 5 to 236 patients. Across all included studies, a total of 90 patients received cilta-cel in the OP setting. Clinical evidence informing OP administration was derived primarily from a small number of retrospective studies, with supportive insights on health-economic outcomes and operational feasibility from economic analyses and survey-based evidence; clinical trials contributed limited OP data.

Most studies (n = 35) were conducted in the United States, whereas the remaining studies were predominantly multinational, involving centers across North America, Europe, Asia, and the Middle East. [10,23,24,25,26,27,28,29,30,31,32,33]. Six studies (five retrospective and one prospective) did not specify their study locations [34,35,36,37,38,39].

One retrospective study evaluated cilta-cel administration exclusively in the OP setting [40]. In addition, across the CARTITUDE-1, -2, and -4 trials, two of the 372 patients received cilta-cel in the OP setting [26,27]. Eleven studies reported data for an IP setting [8,10,24,25,26,27,33,41,42,43,44], and six studies included both IP and OP cohorts [45,46,47,48,49,50]. Thirty-seven studies did not report the setting in which cilta-cel was administered [12,13,28,29,30,34,35,36,37,38,39,51,52,53,54,55,56,57,58,59,60,61,62,63,64,65,66,67,68,69,70,71,72,73,74,75]. These are referred to as the ‘unspecified setting’ throughout the results. Furthermore, one survey explored clinician and administrator perspectives on OP administration of cilta-cel in patients with RRMM [17].

Of all the eligible studies, 35 reported efficacy outcomes, including response rates (n = 29) and survival outcomes (n = 27), of which only three retrospective studies presented data for cilta-cel use in the OP setting [40,45,49] (Table 1). Thirty-five studies reported data on AEs, including CRS and neurologic toxicity, of which four studies presented data in the OP setting [40,45,49,76,77] (Table 1). Only two clinical trials and one retrospective study reported QoL outcomes; the clinical trials were limited to the IP setting, while the retrospective study did not report the administration setting and no QoL data were available for the OP cohorts [12,78,79,80,81,82,83]. Nine studies reported costs or reimbursement amounts associated with cilta-cel, of which three included OP-specific cost data [46,47,48,50,51,61,62,63,69] (Table 1). Similarly, nine studies examined HCRU [13,30,34,41,67,69]. Of these, three studies reported outcomes for OP cohorts [40,45,49] (Table 1).

Considerable variability was observed across studies in patient characteristics such as age, sex, performance status, number of prior treatment lines and other clinical factors. Details of all included studies and patient characteristics are shown in Tables S5 and S6.

### 3.3. Clinical Outcomes

#### 3.3.1. Efficacy: Response and Survival Outcomes

Twenty-nine studies reported response outcomes associated with the use of cilta-cel. The ORRs for cilta-cel were consistently high across administration settings. Patients managed in the OP setting achieved an ORR of 95%, with CR and PR rates of 53% and 43%, respectively [40] (Table 2). IP studies reported a median ORR of 91% (range: 60–100%), with a median CR rate of 79% (range: 35–94%) and median PR rate of 12% (range: 0–25%) [8,10,24,25,26,27,33,41,42,43,44]. In studies with an unspecified setting, median ORR, CR and PR rates were 90% (range: 80–100%), 63% (range: 50–75%), and 28% (range: 17–33%), respectively (Table S7). At 30 days, Gregory et al. reported an ORR of 82% among patients managed in the OP setting, with CR and PR rates of 27% and 55%, respectively [45] (Table 2). Thirty-day response outcomes were not available for IP studies. In the unspecified-setting studies, the median 30-day ORR was 83% (range: 79–94%), with median CR and PR rates of 30% (range: 26–61%) and 53% (range: 33–55%), respectively [30,34,35,58,65] (Table S7).

Twenty-seven studies reported PFS and OS outcomes with use of cilta-cel. However, such data on OP administration of cilta-cel were limited. In OP-focused studies, Waqar et al. 2024 reported survival outcomes, with a 1-year PFS rate of 86% and a 1-year OS rate of 96% [49]. Gregory et al. 2024 observed a 1-year OS rate of 93% and 100% among patients treated in the OP setting and IP setting, respectively; median values were not reached and follow-up duration was not reported [45] (Table 2). In contrast, studies in which cilta-cel was administered exclusively in the IP setting reported a median 1-year PFS of 76% (range: 39–94%) [8,10,24,25,26,27,33,41,42,43,44] and a median 1-year OS of 85% (range: 78–94%) [8,25,26,43,44]. Among unspecified-setting reports, the median 1-year PFS was 68% (range: 24–83%) [13,28,30,58,64,67,74] and the median 1-year OS was 86% (range: 79–91%) [13,30,58,73] (Table S8).

#### 3.3.2. Safety

Thirty-five studies reported safety outcomes for cilta-cel. In the OP studies, the incidence of any-grade CRS was 79% in Ly et al. (2024) and 84% in Gregory et al. (2024) [40,45] (Table 3), which falls within the range reported in IP studies (32–100%), reflecting heterogeneity in patient populations, study design, CRS grading criteria, and monitoring and reporting practices across studies [8,10,24,25,26,27,33,41,42,43,44] (Table S9). In Gregory et al. (2024), the proportion of patients experiencing any CRS event was comparable between OP and IP settings (84% vs. 90%; *p* = 0.413) [45]. Most CRS events in the OP setting were of low grade. In the same study, grade 1 CRS occurred in 45.9% of patients, grade 2 in 35.1%, grade 3 in 2.7%, and no grade 4 events were reported [45]. In Ly et al. (2024), grade 1/2 CRS occurred in 75.0% of patients and grade 3/4 in 4.0% [40]. CRS grading criteria were inconsistently reported across studies; ASTCT criteria were used in some studies where specified, while others did not clearly report the grading framework. The median time to CRS onset in the OP cohort was about 6 days post-infusion, with a median duration of approximately 2 days [40] (Table 3). In comparison, IP studies reported median CRS onset at 6–9 days and a duration of 2.5–9 days [8,10,24,25,26,27,33,41,42,43,44] (Table S9). Among unspecified-setting reports, the median time to CRS onset ranged from 2 to 7 days [13,30,35,64]. In the one patient treated in an OP setting in cohort A of the CARTITUDE-2 trial, grade 2 CRS occurred 9 days after cilta-cel infusion, which resolved after 2 days [76]. Among the 18 patients who experienced grade 1–2 CRS events in Ly et al. (2024), one was monitored in the OP setting without requiring intervention, one required a 2-day hospitalization without specific therapy, and the remaining patients were managed with tocilizumab and/or anakinra [40].

In patients treated with cilta-cel in an OP setting, ICANS was reported in 17% of patients in Ly et al. (2024) with a median time to onset of 8 days (range: 6–14), which lasted for a median of 1 day (range: 1–3) [40]. In Gregory et al., the proportion of patients experiencing ICANS was numerically lower in the OP cohort compared with the IP cohort (21.6% vs. 35%; *p* = 0.237), although the analysis was limited by small cohort sizes and was not powered to detect differences between settings [45] (Table 3). In the OP cohort, ICANS events were predominantly low grade, with grade 1 or 2 events comprising 100% and 88% of cases in Ly et al. (2024) and Gregory et al. (2024), respectively [40,45] (Table 3). Across studies evaluating cilta-cel in the IP setting only, the median incidence of ICANS was 17% (range: 5–23%). The median time to ICANS onset ranged from 7 to 10 days post-infusion, with a median duration ranging from 1 to 7 days [8,10,24,25,26,27,33,41,42,43,44]. In the unspecified-setting studies, the median incidence of ICANS was 15% (range: 4–47%). The median time to ICANS onset ranged from 6 to 8 days post-infusion, with a median duration of approximately 2 days [13,30,34,35,36,37,38,54,57,58,60,64,70,71,72] (Table S9).

In Waqar et al. (2024), the median value of maximum CRS and ICANS grades among outpatients were 1 (range, 0–2) and 0 (range, 0–3), compared with 4 (range, 1–5) and 0 (range, 0–2) among inpatients [49] (Table 3).

Neurologic events, including cranial nerve palsy and parkinsonism, were infrequently reported. These events were primarily reported in IP studies, where longer follow-up enabled detection of these late-onset toxicities that typically emerged 3 to 4 weeks post-infusion. Ly et al. (2024) reported five neurologic events among 24 OP-treated patients (21%; grade 2 in two [8%] patients and grade 3 in three [13%] patients), with a median time to onset of 28 days (range: 12–295 days) [40]. These events were primarily managed with dexamethasone and anakinra [40] (Table 3). The five cases included one unilateral brachial plexus neuritis (grade 3) on day +12 that improved with corticosteroids; two bilateral facial paralysis on days +16 and +28 (both grade 3), of which the first improved following dexamethasone and anakinra with residual jaw numbness, while the second resolved by day +130 with physical therapy; one parkinsonism (grade 2) on day +50 that resolved completely after a 4-day course of dexamethasone; and one unilateral foot drop (grade 2) on day +295, with no treatment reported [40]. In the one patient treated in an OP setting in cohort A of the CARTITUDE-2 trial, grade 2 isolated facial paralysis occurred 29 days after cilta-cel infusion, which resolved completely within 51 days following dexamethasone therapy [76]. The frequency of non-ICANS neurologic events varied across studies. Cranial nerve palsies were reported in a median of 8% patients (range: 1–18%), whereas parkinsonism occurred less frequently, with a median reported frequency of 1% [range: 0–6%]) [8,10,13,26,27,33,40,71] (Table S9).

Other adverse events did not show clear differences in reported rates across settings. One OP study reported grade 1–2 hemophagocytic lymphohistiocytosis/immune effector cell-associated hemophagocytic syndrome (HLH/IEC-HS) in two (8.3%) patients [40] (Table 3). The grade 1 IEC-HS was managed with anakinra alone, whereas grade 2 IEC-HS required treatment with both anakinra and ruxolitinib [40]. The incidence of IEC-HS was 6% among patients treated in an IP setting [44], whereas, in studies that did not report the treatment setting, IEC-HS occurred in 2–3% patients [13,35,71] (Table S9).

### 3.4. Economic Outcomes

#### 3.4.1. Healthcare Resource Utilization

Nine studies examined HCRU for cilta-cel across both IP and OP settings, of which three reported outcomes exclusively for OP cohorts [13,30,34,40,41,45,49,67,69]. Overall, OP administration of cilta-cel was associated with fewer post-infusion hospitalizations, lower ICU utilization, and shorter lengths of stay compared with IP treatment. Two retrospective OP studies reported that 86% and 93% of patients eventually required hospitalization, most often for manageable CRS or MM-related complications. However, these admissions were generally short and anticipated as part of structured OP pathways with predefined escalation to IP care [40]. In one study, three patients (11.1%) who received OP administration required a second hospital admission within 30 days of CAR-T infusion (two for ICANS and one for infection) [49] (Table 4). ICU admission was reported in 7% of OP-treated patients in Ly et al. (2024) [40]. Gregory et al. (2024) reported an overall ICU admission rate of 23% in a combined cohort of OP and IP patients; setting-specific rates were not available [45].

Waqar et al. (2024) evaluated cilta-cel in patients with RRMM across OP, IP and scheduled OP-to-IP cohorts, reporting a median hospital stay of 4 days (range, 0–33) for OP, 19 days (range, 9–36) for IP, and 11 days (range, 4–69) for OP-to-IP patients [49]. Gregory et al. (2024) reported a median length of stay of 6 days (interquartile range [IQR], 3–15) across OP and IP settings [45], while Ly et al. (2024) reported a median hospitalization duration of 6.5 days (range, 2–11) in the OP setting [40]. Length-of-stay comparisons across studies may be confounded by institutional practices related to admission and discharge (Table 4).

In the Wesson et al. (2024) study, in which all cilta-cel recipients were treated in the IP setting, prolonged hospitalization within 30 days of infusion was reported in approximately 21% of patients [41]. ICU admission in the unspecified-setting cohorts occurred in 8–22%, patients and the median length of hospital stay ranged from 11 to 17 days [13,34] (Table S10). Overall, while unplanned admissions are common in OP cohorts, the total length of stay and intensity of resource use were lower in OP settings compared with IP care.

#### 3.4.2. Cost Outcomes

Nine studies reported costs or reimbursement amounts associated with CAR-T administration across IP and OP settings, of which three specifically evaluated the economic impact of administering cilta-cel in the OP setting [46,47,48,50,51,61,62,63,69]. Jagannath et al. estimated per-patient savings of approximately $19,000 when cilta-cel was delivered in the OP versus IP setting, driven primarily by reduced peri-infusion IP hospitalization and administration costs [48] (Table 5). In a cost-per-responder (CPR) modeling analysis, cilta-cel was associated with cost savings compared with real-world SOCs in the base case scenario where cilta-cel was administered exclusively in an IP setting. In an alternate scenario, where 30% of cilta-cel administration was assumed to occur in an OP setting, cilta-cel was found to result in an additional cost saving of $7328 per complete responder and $217 per PFS-month [46,50]. In a similar CPR analysis based on the CARTITUDE-4 trial, Hansen et al. (2024) and Hansen et al. (2023) reported additional savings of $7598 per complete responder and $294 per PFS-month, reflecting lower infusion and monitoring costs under OP administration assumptions [46,50]. Details of cost outcomes from the included studies are presented in Table S11. A real-world claims analysis, in which the treatment setting was not reported, estimated mean all-cause healthcare costs of $132,051 per patient per month (PPPM) for cilta-cel administration [69]

## 4. Discussion

This systematic review aimed to summarize current evidence on the efficacy, safety, HCRU, costs and QoL associated with cilta-cel CAR-T therapy in OP, IP or both settings. Interpretation of these findings should be cautious, as most OP data derive from small, retrospective, and non-comparative studies.

This systematic review found that cilta-cel was associated with high response rates and survival outcomes in patients with RRMM treated in OP, IP or both settings, with a manageable safety profile. The high response rates reported across OP cohorts are consistent with those achieved in IP studies, including the pivotal CARTITUDE trials [8,10,24,25,26,27,33,41,42,43,44]. Across studies with the OP data available, 12-month PFS and OS outcomes (approximately 86% and 96%, respectively) [49] were within the range of outcomes reported in IP studies [8,10,24,25,26,27,33,41,42,43,44]. These findings support the feasibility of OP infusion in selected patients. However, they do not establish equivalence between OP and IP settings because comparisons are indirect, cohorts are small, and patient selection and institutional practices likely differ.

The safety profile of cilta-cel in OP studies was consistent with that observed in IP settings [8,10,24,25,26,27,33,41,42,43,44]. The incidence and timing of acute adverse events, including CRS and ICANS, were within the ranges historically reported in clinical trials with IP administration of cilta-cel [8,10,24,25,26,27,33,41,42,43,44]. However, these observations are based on descriptive comparisons across heterogeneous studies and should be interpreted cautiously. This reflects the fact that CRS and ICANS with cilta-cel tend to have a predictable time course, particularly the several-day delay in CRS onset, which can be accommodated in an OP setting with adequate monitoring. In clinical practice, centers administering OP CAR-T may employ daily check-ins during the first 7 days post-infusion, after which check-ins become optional, along with remote monitoring technologies and quick admission pathways to effectively manage such delayed toxicities [84]. Review of the included studies indicated that the post-infusion care following OP administration was comparable to IP care in managing low rates of severe ICANS and CRS. Non-ICANS neurologic events, such as cranial nerve palsies, were infrequent and typically occurred beyond the first 2–3 weeks post-infusion, while parkinsonism was reported in a very low proportion of patients across studies. These events were managed with close monitoring and immunosuppressive therapy, and their incidence appears unrelated to the initial care setting [40,76]. Overall, current evidence suggests that vigilant monitoring enables safe OP administration without increasing patient risk, even beyond the early post-infusion period.

Adopting OP administration of cilta-cel for myeloma could provide substantial benefits in improving both health system efficiency and patient QoL [40,42,45,49]. An expert roundtable on cilta-cel emphasized that OP administration can effectively reduce the burden on hospital resources and has the potential to expand access to therapy by mitigating delays linked to limited bed availability, without compromising patient safety or treatment efficacy, suggesting a promising model that requires further validation [16]. Consistent with these observations, the current review identified numerically shorter hospitalization durations and less ICU utilization in an OP setting. From the patient perspective, recovery in an ambulatory environment may improve comfort, autonomy, and QoL [18]. However, OP-specific QoL data for cilta-cel are currently lacking and available evidence on QoL outcomes is limited to IP-treated populations. Although direct QoL data from OP cohorts are lacking, cilta-cel recipients in IP trials have reported HRQoL improvements over standard of care [78,79,80,81,82,83], suggesting that these benefits could extend or even be amplified in OP settings where hospitalization is minimized.

Economic considerations further strengthen the rationale for OP administration of cilta-cel. Shifting appropriate patients to OP administration reduces treatment-related costs and resource demands, without compromising clinical outcomes, as evidence from clinicians and administrators indicates that OP cilta-cel can be delivered safely while supporting institutional sustainability and reducing patient burden [17]. The analyses by Jagannath et al. estimated significant per-patient savings when hospitalization is avoided, and these benefits scale substantially as utilization increases [85]. Our review also highlighted that IP CAR-T remains exceedingly costly, while OP cilta-cel administration was associated with additional cost savings [46,50]; thus, even modest reductions in hospital days or ICU admissions per patient may yield meaningful system-wide cost savings. However, it should be noted that these findings were based on scenario-based economic models conducted from a US payer perspective and may vary substantially by healthcare system, reimbursement, and institutional pathways. Therefore, these results should be interpreted as context-specific rather than broadly generalizable. This has implications for payers and institutions, as these efficiencies make CAR-T a more sustainable therapy while potentially broadening access by freeing hospital beds for patients who truly require IP care. Economic considerations and improved access are thus major incentives driving the ongoing transition to OP CAR-T programs.

To translate these benefits broadly, careful planning and infrastructure are essential. Experienced centers recommend standardized protocols for patient selection (e.g., absence of severe cardiopulmonary comorbidities or rapidly progressive disease), structured caregiver support, and clear escalation pathways for adverse event management [16,86]. A qualitative study of clinicians and administrators underscored that, while OP CAR-T substantially decreases IP burden, it also introduces challenges such as caregiver preparedness, emergency readiness, and institutional coordination [17]. These findings underscore that safe and effective OP CAR-T delivery depends not only on clinical protocols but also on integrated systems of support, streamlined workflows, and robust communication across care settings. Many institutions begin with a small subset of low-risk patients and expand eligibility as confidence and expertise grow. Training nursing and OP staff to recognize early CRS/ICANS symptoms, coupled with coordination with emergency services, further strengthens safety [86]. When these elements are in place, existing data support the feasibility of OP cilta-cel administration, although conclusions regarding effectiveness remain preliminary.

These findings align with prior systematic reviews across hematologic malignancies, which also demonstrated comparable response and toxicity profiles between OP and IP cohorts [14]. Together, this evidence supports the hypothesis that cilta-cel can be effectively delivered in an OP setting, offering additional logistical and economic advantages while maintaining consistent clinical outcomes and a manageable safety profile.

Future research may also explore comparative evaluations of CAR-T therapy, including cilta-cel, against other immune-based treatments for RRMM, such as bispecific antibodies and antibody–drug conjugates, to better contextualize differences in efficacy, safety, quality of life, and healthcare resource utilization.

Despite these promising findings, several limitations must be acknowledged. First, outpatient cilta-cel evidence is derived from small, often single-center studies with limited follow-up durations, and OP-specific efficacy conclusions are based on a small number of retrospective analyses and very limited trial-level data; therefore, findings should be interpreted cautiously. In addition, selection bias is likely, as patients treated in the OP setting often have favorable baseline characteristics and strong social support. This may contribute to better observed safety and efficacy outcomes and may overstate comparability with IP cohorts, thereby limiting generalizability, even though no clear differences across settings were observed [10]. The current evidence was also drawn largely from indirect comparisons, as no head-to-head randomized studies comparing OP versus IP administration have yet been conducted, despite generally consistent trends across published studies. Furthermore, a formal, tool-based assessment of methodological quality or risk of bias was not performed, as this review aimed to provide a descriptive synthesis across a highly heterogeneous evidence base spanning multiple study designs. As a result, the certainty of evidence across outcomes could not be formally graded, and findings were interpreted qualitatively. Long-term outcomes, including durability of response and late toxicities, remain incompletely characterized and warrant further investigation in larger, prospective studies. Although no associations have been established between short-term toxicities (CRS and ICANS) and late-onset neurologic events, suggesting that initial care setting may be unlikely to influence long-term safety, prospective trials directly comparing IP and OP administration are needed to confirm these observations. Until such data mature, our conclusions should be considered preliminary but encouraging.

In summary, the emerging evidence suggests the feasibility of OP administration of cilta-cel in appropriately equipped and experienced centers. With prudent patient selection, standardized monitoring, and coordinated multidisciplinary care, OP CAR-T may achieve encouraging outcomes in selected patient populations. However, given the retrospective, non-comparative nature of the available evidence, these findings should be interpreted cautiously and require confirmation in prospective, multicenter comparative studies.

## 5. Conclusions

Findings from this review suggest that outpatient administration of cilta-cel may be feasible for carefully selected patients, based on evidence from approximately 90 patients treated in OP settings across retrospective studies. While CAR-T therapies are typically administered in an IP setting, OP administration has the potential to reduce economic burden without clear signals of compromised clinical outcomes. Prospective, multicenter comparative studies are needed to confirm long-term safety, durability of response, QoL outcomes, and cost-effectiveness before OP CAR-T administration can be considered for broader implementation.

## Figures and Tables

**Table 1:** Studies reporting outcomes for OP administration of cilta-cel.

Study	Country	Study Design	Patient Population	Setting (IP/OP/Both)	Sample Size, N	Age, Median (Years)	Male (%)	ECOG PS (%)	Number of Prior Lines	Prior Transplant Therapy, (%)	List of Outcomes
Ly (2024) [40]	USA	Retrospective study	RRMM (5 L+)	OP	24	59 (38–79)	62.0	NR	8 (4–15)	Overall-SCT: 63 (one pt had both allo & auto-SCT) Auto-SCT: 54.1 Allo-SCT: 12.5	Response, AEs, hospitalization and ICU stay
Waqar (2024) [49]	USA	Retrospective study	RRMM (5 L+)	OP	27	61 (46–76)	44.4	NR	5 (4–10)	Auto-SCT: 88.9	PFS, OS, AEs and hospitalization
				IP	6	62 (41–76)	83.3	NR	5 (4–7)	Auto-SCT: 33.3	
				Scheduled OP–IP	10	58.5 (38–75)	40.0	NR	6 (4–11)	Auto-SCT: 90.0	
Gregory (2024) [45]	USA	Retrospective study	RRMM	OP	37	NR	NR	NR	NR	Prior auto transplant: 37.8	Response, AEs, LOS and ICU stay
				IP	20	NR	NR	NR	NR	Prior auto transplant: 60.0	
J&J Medical Connect report (2025) [76]	Multinational	Clinical trial (CARTITUDE-2 cohort A and B)	Len refractory MM (1–3 L)	OP	2 ^a^	NR	NR	NR	2 (1–3)	NR	AEs
Jagannath (2023) [48]	USA	Economic study	RRMM (3 L+)	Both (OP, IP)	NR	NR	NR	NR	NR	NR	Costs (IP, OP, diagnostics, drug acquisition and administration, procedures), HCRU outcomes
Hansen (2024) [46]	USA	Economic study	Len refractory MM	Both (OP, IP)	NR	NR	NR	NR	NR	NR	Total cost per treated patient, total cost per CR, cost per month in PFS
Hansen (2023) [50]	USA	Economic study	RRMM	Both (OP, IP)	NR	NR	NR	NR	NR	NR	Cost per responder. Drug acquisition, administration, healthcare resource use, and monitoring costs

Abbreviations: AEs, adverse events; allo-SCT, allogeneic stem cell transplant; auto-SCT, autologous stem cell transplant; cilta-cel, ciltacabtagene autoleucel; CR, complete response; ECOG PS, Eastern Cooperative Oncology Group performance status; HCRU, healthcare resource utilization; ICU, intensive care unit; IP, inpatient; Len refractory MM, lenalidomide-refractory multiple myeloma; LOS, length of stay; NR, not reported; OP, outpatient; OS, overall survival; PFS, progression-free survival; RRMM, relapsed/refractory multiple myeloma. ^a^ Only two patients received cilta-cel in the OP setting.

**Table 2:** Response and survival outcomes for cilta-cel in RRMM patients treated in OP setting.

Study	Study Design	Follow-Up, Months	N	ORR %	CR %	PR %	OS	PFS	Other Details
Gregory (2024) [45]	Retrospective study	NA	OP: 11	30-day: 82	30-day: 27	30-day: 55	Median: NR 12-mo OS: 93	-	46% had ≥ VGPR
			IP: 20	-	-	-	Median: NR 12-mo OS: 100	-	-
Ly (2024) [40]	Retrospective study	4.6	OP: 21	95	53	43	-	-	86.0% had ≥ VGPR
Waqar (2024) [49]	Retrospective study	7.7	OP: 27	-	-	-	Median: NR 12-mo OS: 96%	Median: NR 12-mo OS: 86%	-
			Scheduled OP–IP: 10	-	-	-	OS was similar across the administration types	PFS was similar across the administration types	-
			IP: 6	-	-	-			-

Abbreviations: CR, complete response; IP, inpatient; OP, outpatient; ORR, overall response rate; OS, overall survival; PFS, progression-free survival; PR, partial response; sCR, stringent complete response; VGPR, very good partial response.

**Table 3:** Safety data for cilta-cel in RRMM patients treated in OP setting.

Study	Study Design	Follow-Up, Months	N	CRS	ICANS	NEs (Excluding Parkinsonism)	Parkinsonism	HLH/IEC-HS
Ly (2024) [40]	Retrospective Study	4.6	24	Any grade: 79.2% Grade 1/2: 75.0% Grade 3/4: 4.0% Median (range) time to onset: 6 (0–9) days Median (range) duration: 2 (1–6) days	Overall: 17.0% inferred based on abstract Grade 3/4: 0.0% Median (range) time to onset: 8 (6–14) days Median (range) duration: 1 (1–3) days	Any grade: 20.8% Grade 2: 8.0% Grade 3/4: 13.0%	Grade 2: Parkinsonism (secondary to CAR-T): 4%	Grade 1/2: 8.3%
						Grade 2: Unilateral foot drop Grade 3: Unilateral brachial plexus neuritis, bilateral facial paralysis, bilateral facial paralysis		
Waqar (2024) [49]	Retrospective study	7.7	OP: 27	Median value of maximum grade: 1 (0–2) Grade >= 3: 0	Median value of maximum grade: 0 (0–3)	NR	NR	NR
			Scheduled OP–IP: 10	Median value of maximum grade: 1 (0–2)	Median value of maximum grade: 0 (0–0)	NR	NR	NR
			IP: 6	Median value of maximum grade: 4 (1–5)	Median value of maximum grade: 0 (0–2)	NR	NR	NR
Gregory (2024) [45]	Retrospective study	NR	OP: 37	Any grade: 83.8	Any grade: 21.6	NR	NR	NR
				Grade 1: 45.9 Grade 2: 35.1 Grade 3: 2.7 Grade 4: 0	Grade 1: 16.2 Grade 2: 2.7 Grade 3: 0 Grade 4: 2.7			
				*p*-value = 0.413	*p*-value = 0.237			
			IP: 20	Any grade: 90	Any grade: 35	NR	NR	NR
				Grade 1: 50 Grade 2: 20 Grade 3: 10 Grade 4: 10	Grade 1: 20 Grade 2: 5 Grade 3: 0 Grade 4: 10			
J&J Medical Connect report (2025) [76]	Clinical trial (CARTITUDE-2 cohort A)	-	1 ^a^	Grade 2	-	Grade 2: Facial paralysis	NR	NR

Abbreviations: CRS, cytokine release syndrome; ICANS, immune effector cell-associated neurotoxicity syndrome; NEs: neurologic events; NR, not reported; OP, outpatient. ^a^ Only one patient (of the 20) received cilta-cel in OP setting.

**Table 4:** HCRU data for cilta-cel in RRMM patients treated in OP setting.

Study	Study Design	Tx Setting	N	Hosp. Rate, n (%)	LOS, Median (Range)	ICU Admi. (%)	Reason for Hosp.
Waqar (2024) [49]	Retrospective study	OP	27	92.6	4 days (0–33)	NR	CRS: 81.5%. Other: 18.5%
				30 d readmission: 11.1			Reasons for readmission (2 for ICANS, 1 for infection)
		IP	6	100	19 days (9–36)	NR	MM-related: 66.7%; AE: 16.7%. Other: 16.7%
		Scheduled OP–IP	10	100	11 days (4–69) Day 1 *		
Ly (2024) [40]	Retrospective study	OP	14	86	6.5 days (2–11) 5 days (0–9) *	7	AE: 79%. Other: 21%
Gregory (2024) [45]	Retrospective study	Both (OP, IP)	57-OP:37, IP: 20	NR	6 days (IQR: 3–15)	23	NR

Abbreviations: AE, adverse event; CRS, cytokine release syndrome; ICU, intensive care unit; IP, inpatient; IQR, interquartile range; LOS, length of stay; MM, multiple myeloma; NR, not reported; OP, outpatient; Tx, treatment. * Time to hospitalization.

**Table 5:** Cost data for cilta-cel in RRMM patients treated in OP setting.

Study	Study Design	Tx	Tx Setting	Cost Elements Modeled	Time Horizon	Cost Year	Total Costs	Pre-Infusion Costs	Peri-Infusion Costs	Monitoring Costs (Lab and Diagnostics, Adverse Event Management Costs, etc.)
Jagannath (2023) [48]	Retrospective study	Cilta-cel	OP	Infusion costs, diagnostics, procedures, AE management costs	1 year	2021 USD	$142,012	18,268	4232	119,511
			IP				$160,933		23,154	
Hansen (2024) [46]	Cost-per-responder analysis	Cilta-cel	Both (OP, IP)	Drug acquisition, administration, healthcare resource use, and monitoring costs	NR	2024 USD	Base case: 100% IP Cost per CR: $1,027,451; CPM in PFS: $25,122 Scenario analysis: 70% IP, 30% OP Cost per CR: $1,020,123; CPM in PFS: $24,905	NR	NR	NR
		SoC^a^					Cost per CR: $4,636,587; CPM in PFS: $37,255			
Hansen (2023) [50]	Cost-per-responder analysis	Cilta-cel	Both (OP, IP)	Drug acquisition, administration, healthcare resource use, and monitoring costs	NR	2023 USD	Base case: 100% IP Cost per CR: $981,884; CPM in PFS: $31,555 Scenario analysis: 70% IP, 30% OP Cost per CR: $974,286; CPM in PFS: $31,261	NR	NR	NR
		SoC^b^					Cost per complete responder: $1,041,171; Cost per CPM in PFS: $4,776,015			

Abbreviations: CPM, cost per month; CR, complete response; IP, inpatient; NR, not reported; OP, outpatient; PFS, progression-free survival; SoC: standard of care; Tx, treatment; USD, United States Dollar. SoC^a^: daratumumab + pomalidomide + dexamethasone (25.1%), daratumumab + carfilzomib + dexamethasone (22.8%), bortezomib + dexamethasone (15.4%), daratumumab + bortezomib + dexamethasone (12.9%), carfilzomib + dexamethasone (11.9%), pomalidomide + dexamethasone (5.4%), selinexor + bortezomib + dexamethasone (4.8%), and elotuzumab + pomalidomide + dexamethasone(1.8%). SoC^b^: daratumumab plus pomalidomide and dexamethasone [DPd] or pomalidomide plus bortezomib and dexamethasone [PVd].

## Data Availability

All data generated or analyzed during this study are included in this published article (and its Supplementary Information Files).

## Supplementary Materials

The following supporting information can be downloaded at: https://www.mdpi.com/article/10.3390/cancers18050755/s1, Table S1: Embase search strategy; Table S2. Medline search strategy; Table S3. Cochrane search strategy; Table S4. Eligibility criteria; Table S5. Characteristics of included studies; Table S6. Patient characteristics; Table S7. Response results for cilta-cel in RRMM patients treated in IP or unspecified treatment setting; Table S8. Survival outcomes for cilta-cel in RRMM patients treated in IP or unspecified treatment setting; Table S9. Safety outcomes for cilta-cel in RRMM patients treated in IP or unspecified treatment setting; Table S10. HCRU outcomes for cilta-cel in RRMM patients treated in IP or unspecified treatment setting; Table S11. Cost outcomes for cilta-cel in RRMM patients treated in IP or unspecified treatment setting; Table S12. Summary of databases and online platforms searched.
