# Supplementary material for: Outpatient Versus Inpatient Administration of Ciltacabtagene Autoleucel in Multiple Myeloma: A Systematic Review of Clinical, Economic, and Humanistic Outcomes
Source: Cancers (Basel). 2026 Feb 26;18(5):755. doi: 10.3390/cancers18050755 (PMC12984309; doi:10.3390/cancers18050755)
Supplement: Supplementary file 1 [file cancers-18-00755-s001.zip › cancers-4111911-supplementary.pdf]

cancers

MDPI

Supplementary materials: Outpatient Versus Inpatient Administration of Ciltacabtagene Autoleucel in Multiple Myeloma: A Systematic Review of Clinical, Economic, and Humanistic Outcomes

Tara Gregory, Kevin C De Braganca, Victoria Alegria, Matthew Perciavalle, Ravi Potluri, Sandip Ranjan, Todd Bixby and Zaina P. Qureshi

Table S1. Embase search strategy.

#	Query	# hits
1	exp multiple myeloma/	115,100
2	(myeloma* or (multiple adj myeloma*) or plasmacytom* or plasmocytom* or (monoclonal adj gammopath*)).ti,ab.	129,619
3	1 or 2	153,000
4	exp ciltacabtagene autoleucel/	891
5	(Carvykti or Ciltacabtagene* or LCAR B38M or LCAR-B38M or jnj 68284528 or jnj 4528 or Jnj4528).mp.	939
6	4 or 5	939
7	3 and 6	815
8	exp animals/ not humans/	5,961,772
9	(comment or letter or editorial or "case reports").pt.	2,213,488
10	(case stud$ or case report$).ti.	540,473
11	(address or autobiography or biography or case reports or veterinary trials or veterinary as topic or comment or dictionary or directory or duplicate publication or editorial or festschrift or guideline or historical article or interactive tutorial or interview or lecture or legislation or letter or observational study, veterinary or patient education handout or personal narrative or practice guideline or editorial or erratum or letter or note or review or short survey or comment*).pt.	7,338,901
12	8 or 9 or 10 or 11	13,550,439
13	7 not 12	567
14	limit 13 to (human and English language)	564
15	limit 14 to 05 August 2025	**548**

Table S2. Medline search strategy.

#	Query	# hits
1	exp multiple myeloma/	51,165
2	(myeloma* or (multiple adj myeloma*) or plasmacytom* or plasmocytom* or (monoclonal adj gammopath*)).ti,ab.	77,976
3	1 or 2	85,776
4	(Carvykti or Ciltacabtagene* or LCAR B38M or LCAR-B38M or jnj 68284528 or jnj 4528 or Jnj4528).mp.	171
5	3 and 4	145
6	exp animals/ not humans/	5,363,132
7	(comment or letter or editorial or "case reports").pt.	4,605,764
8	(case stud$ or case report$).ti.	454,891
9	(address or autobiography or biography or case reports or veterinary trials or veterinary as topic or comment or dictionary or directory or duplicate publication or editorial or festschrift or guideline or historical article or interactive tutorial or interview or lecture or legislation or letter or observational study, veterinary or patient education handout or personal narrative or practice guideline or editorial or erratum or letter or note or review or short survey or comment*).pt.	8,237,374
10	6 or 7 or 8 or 9	13,401,327
11	5 not 10	88

12	limit 11 to (human and English language)	86
15	limit 12 to 2025Aug05	**66**

Table S3. Cochrane search strategy.

#	**Query**	# **hits**
#1	[mh "Multiple Myeloma"]	2,509
#2	(myeloma* or (multiple NEAR/2 myeloma*) or plasmacytom* or plasmocytom* or (monoclonal NEAR/2 gammopath*)):ti,ab (Word variations have been searched)	7,071
#3	#1 or #2	7,283
#4	(Carvykti or Ciltacabtagene* or "LCAR B38M" or LCAR-B38M or "jnj 68284528" or "jnj 4528" or Jnj4528):ti,ab (Word variations have been searched)	50
#5	#3 and #4	50
#6	[mh "animals"] NOT [mh "humans"]	1,307
#7	(comment or letter or editorial or "case reports"):pt (Word variations have been searched)	0
#8	(case stud$ or case report$):ti (Word variations have been searched)	893
#9	(address or autobiography or biography or case reports, veterinary trials, veterinary as topic or comment or dictionary or directory or duplicate publication or editorial or festschrift or guideline or historical article or interactive tutorial or interview or lecture or legislation or letter or observational study, veterinary or patient education handout or personal narrative or practice guideline or review or editorial or erratum or letter or note or short survey or comment*):pt (Word variations have been searched)	7,455
#10	#6 or #7 or #8 or #9	9655
#11	#5 not #10	50
#12	#11 with Cochrane Library publication date till Aug 2025	**39**

Table S4. Eligibility criteria.

**Category**	**Inclusion criteria**	**Exclusion criteria**
Population	Patients with multiple myeloma	• Non-human studies
Intervention	Ciltacabtagene autoleucel (cilta-cel)	Interventions other than cilta-cel
Comparators	Any	Not applicable
Outcomes	Studies reporting any relevant efficacy, safety, economic, HCRU and humanistic outcomes including:<br>• Efficacy: ORR, CR, PR, PFS, OS etc.<br>• Safety: CRS, ICANS, non-ICANS/neurologic events etc.<br>• Costs: direct medical costs, hospitalization costs, OP care costs etc.<br>• HCRU: hospitalizations, ICU admissions, OP visits, length of stay etc.<br>• Humanistic outcomes: QoL/HRQoL etc.	Studies not reporting any of the relevant outcomes
Study types	Any study design, including:<br>• Interventional studies<br>• Observational studies (prospective, retrospective, surveys)<br>• Economic models	• Case studies/reports, case series, protocols, validation studies<br>• Comments, editorials, magazine, letter to editor, expert opinions, books, errata<br>• Systematic reviews, meta-analyses and narrative reviews were excluded (but were flagged for bibliographic checking and their reference lists were checked to identify studies that might not have been indexed in databases)
Language	Studies published in English	Non-English studies

Abbreviations: CAR-T, chimeric antigen receptor T-cell; CR, complete response; CRS, cytokine release syndrome; Cilta-cel, ciltacabtagene autoleucel; HCRU, health care resource use; HRQoL, health-related quality of life; ICANS, immune effector cell-associated neurotoxicity syndrome; ICU, intensive care unit; IP, inpatient; MM, multiple myeloma; OP, outpatient; ORR, overall response rate; OS, overall survival; PFS, progression-free survival; PR, partial response; QoL, quality of life.

Table S5. Characteristics of included studies.

Study	Country	Trial name/ID	Study design	Treatment	Patient population	Setting (IP/OP/both)	Sample size, N	List of outcomes
Mi (2023) [42]	China	CARTIFAN-1/NCT03758417	Phase II trial	Cilta-cel	RRMM (4L+)	Inpatient	48	Response, TTR, DOR, PFS, OS, and AEs
Xu (2024) [43]	China	LEGEND-2/NCT03090659	Phase I trial	Cilta-cel	RRMM (3L+)	Inpatient	74	Response, TTR, DOR, PFS, OS, and AEs
Berdeja (2021) [8]	USA	CARTITUDE-1/NCT03548207	Phase Ib/II	Cilta-cel	RRMM (4L+)	Inpatient	97	Response, TTR, DOR, PFS, OS and AEs
Martin (2022) [80]	USA	CARTITUDE-1/NCT03548207	Phase II	Cilta-cel	RRMM (4L+)	Inpatient	68	HRQoL
Hillengass (2022) [27]	Multinational	CARTITUDE-2 Cohort A/NCT04133636	Phase II trial	Cilta-cel	Len refractory MM after 1-3 prior LOT	Inpatient	20 (1 OP)*	Response, TTR, DOR, PFS, OS, and AEs
Cohen (2024) [25]	Multinational	CARTITUDE-2 Cohort A expansion subgroup/ NCT04133636	Phase II trial	Cilta-cel	Len refractory MM after 1-3 prior LOT	Inpatient	23	Response, TTR, DOR, PFS, OS, and AEs
Hillengass (2023) [26]	Multinational	CARTITUDE-2 Cohort B/NCT04133636	Phase II trial	Cilta-cel	MM (2L); PI and IMiD exposed; No prior Tx with CAR-T/anti-BCMA therapies	Inpatient	19 (1 OP)*	Response, TTR, DOR, PFS, OS, and AEs
Cohen (2022) [23,24]	Multinational	CARTITUDE-2 Cohort C/NCT04133636	Phase II trial	Cilta-cel	RRMM (previously treated incl BCMA-targeting agent)	Inpatient	20	Response, TTR, DOR, PFS, OS, and AEs
Bertrand (2024) [33]	Multinational	CARTITUDE-2 Cohort D/NCT04133636	Phase II trial	Cilta-cel	NDMM who achieved < CR after ASCT front line therapy	Inpatient	17	Response, TTR, DOR, PFS, OS, and AEs
San-Miguel (2023) [10]	Multinational	CARTITUDE-4/NCT04181827	Phase III RCT	Cilta-cel, SoC	Len refractory MM after 1-3 prior LOT	Inpatient	416 (208 apheresis vs 208 SOC) 176 (cilta-cel) 208 (SoC)	Response, TTR, DOR, PFS, OS, and AEs, HRQoL
Wu (2023) [51]	China	-	Economic study	Cilta-cel	RRMM (4L+)	NR	NR	Cost effectiveness analysis- Chinese healthcare system
Hansen (2024) [47]	Multinational	-	Economic study	Cilta-cel	RRMM	Both	NR	Cost per responder
Gill (2023) [52]	USA	-	Retrospective study	Cilta-cel/Ide-cel	RRMM (5L+)	NR	53 (18 cilta-cel, 35 ide-cel)	Response, DOR, PFS, OS, and AEs
Kocoglu (2023) [53]	USA	-	Retrospective study	Cilta-cel/Ide-cel	RRMM (9-11 LOT)	NR	23 (12 cilta-cel, 11 ide-cel)	Response and AEs

Study	Country	Trial name/ID	Study design	Treatment	Patient population	Setting (IP/OP/both)	Sample size, N	List of outcomes
Christodoulou (2023) [54]	USA	-	Retrospective study	Cilta-cel/Ide-cel	RRMM (5L+)	NR	15	Response and AEs
Vieira (2023) [55]	USA	-	Prospective study	Cilta-cel	RRMM	NR	25	PFS
Attar (2023) [56]	USA	-	Case series	Cilta-cel	RRMM (7L+)	NR	5	Response, PFS, and AEs
Hansen (2023) [57]	USA	-	Retrospective study	Cilta-cel	RRMM	NR	143	Response, PFS, OS, AEs and hospital stay
Hansen (2025) [58]	USA	-	Retrospective study	Cilta-cel/ide-cel	RRMM	NR	586 (Cilta-cel: 236, ide-cel: 350)	Response (CR, PR), PFS, OS, Safety (CRS, ICANS, non-relapse mortality, SPMs)
Sidana (2025) [13]	USA	-	Retrospective study	Cilta-cel	RRMM	NR	236	ORR, CR, OS, PFS, CRS, ICANS, HLH, NT, parkinsonism, mortality, SPM
Schorr (2023) [59]	USA	-	Retrospective study	Cilta-cel/Ide-cel	RRMM	NR	34 (cilta-cel: 9, ide-cel: 25)	AEs
Mejia Saldarriaga (2023) [60]	USA	-	Retrospective study	Cilta-cel/Ide-cel	RRMM	NR	39 (cilta-cel: 25, ide-cel: 14)	AEs
Wesson (2024) [41]	USA	-	Retrospective study	Cilta-cel/Ide-cel	RRMM	Inpatient	129 (cilta-cel: 39, ide-cel: 90)	ORR, CR, NRM, resource utilization, infections, Safety, CRS, infections
Kamboj (2024) [28]	Multinational (5 centers)	-	Retrospective study	Cilta-cel	RRMM	NR	73	Response rate (sCR, CR, PR, VGPR, >=VGPR, ORR, disease progression), OS and AEs
Yamamoto (2024) [61]	USA	-	Economic study	Cilta-cel	RRMM (3L+)	NR	NR	Cost-effectiveness
Kapinos (2023) [62]	USA	-	Economic study	Cilta-cel	RRMM (3L+)	NR	NR	ICER
Agostinho (2025) [63]	Brazil	-	Economic study	Cilta-cel	RRMM	NR	NR	HCRU (Total cost)
Dima (2025) [35]	NR	-	Retrospective study	Cilta-cel	RRMM	NR	105	Response, AEs
Merz (2025) [30]	Multinational	-	Retrospective study	Cilta-cel/Ide-cel	RRMM	NR	204 (cilta-cel: 42, ide-cel: 162)	Response, PFS, OS, AEs, HCRU
Merz (2025) [64]	Germany	-	Retrospective study	Cilta-cel/Ide-cel	RRMM	NR	343 (cilta-cel: 77, ide-cel: 266)	Response, PFS, OS, AEs
Fandrei (2025) [65]	Germany	-	Retrospective study	Cilta-cel/Ide-cel	RRMM	NR	52 (cilta-cel: 18, ide-cel: 34)	Response, AEs
Wiemers (2024) [66]	Germany	-	Retrospective study	Cilta-cel/Ide-cel	RRMM	NR	61 (cilta-cel: 27, ide-cel: 34)	AEs

Study	Country	Trial name/ID	Study design	Treatment	Patient population	Setting (IP/OP/both)	Sample size, N	List of outcomes
Atanackovic (2025) [34]	NR	-	Prospective study	Cilta-cel/Ide-cel	RRMM	NR	39 (cilta-cel: 23, ide-cel: 16)	Response, PFS, AEs
Richardson (2025) [67]	Germany	-	Retrospective study	Cilta-cel/Ide-cel	RRMM	NR	10	Response, PFS, AEs
Waldschmidt (2024) [68]	Germany	-	Retrospective study	Cilta-cel/Ide-cel	RRMM	NR	23 (cilta-cel: 6, ide-cel: 17)	Response
Ailawadhi (2025) [69]	USA	-	Economic study	Cilta-cel/Ide-cel	RRMM	NR	105 (cilta-cel: 47, ide-cel: 58)	HCRU (Inpatient stays (PPPM)) Costs (All-cause total health care costs, medical costs, ambulatory visit costs)
Amoozgar (2024) [70]	USA	-	Retrospective study	Cilta-cel/Ide-cel	RRMM	NR	114 (cilta-cel: 64, ide-cel: 50)	Response, PFS, OS, AEs
Hosoya (2024) [38]	USA	-	Retrospective study	Cilta-cel/Ide-cel	RRMM	NR	59 (cilta-cel: 28, ide-cel: 31)	PFS
Lim (2025) [71]	USA	-	Retrospective study	Cilta-cel	RRMM	NR	235	AEs (CRS, ICANS, CNP, parkinsonism, HLH/IEC-HS, Immune-effector cell enterocolitis)
Lim (2025) [72]	USA	-	Retrospective study	Cilta-cel	RRMM	NR	235	AEs (ICANS, CNP, parkinsonism, Immune-effector cell enterocolitis (IEC-EC), IEC-EC + IEC-CNP, IEC-EC+IEC-parkinsonism)
Ailawadhi (2025) [73]	USA	-	Retrospective study	Cilta-cel	RRMM	NR	182	OS
Fandrei (2024) [36]	NR	-	Retrospective study	Ide-cel	RRMM	NR	34	Response, PFS, AEs
Li (2024) [12]	USA	-	Retrospective study	Cilta-cel/Ide-cel	RRMM	NR	99 (cilta-cel: 50, ide-cel: 49)	HRQOL
Mitch (2025) [44]	USA	-	Retrospective study	Cilta-cel	MM	Inpatient	32	AEs
Artzenroth (2025) [39]	NR	-	Retrospective study	Cilta-cel/Ide-cel	RRMM	NR	45 (cilta-cel: 24, ide-cel: 21)	Response, AEs
Gul (2024) [37]	NR	-	Retrospective study	Cilta-cel	RRMM	NR	172	CRS, NT, OS
Kumar (2024) [29]	Multinational (15 Institutions in 6 countries)	-	Retrospective study	Cilta-cel (4% Ide-cel and 4% investigational CAR-T)	RRMM	NR	52	AEs

Study	Country	Trial name/ID	Study design	Treatment	Patient population	Setting (IP/OP/both)	Sample size, N	List of outcomes
Varga (2024) [74]	USA	-	Retrospective study	Cilta-cel/Ide-cel	RRMM	NR	42 (cilta-cel: 16, ide-cel: 26)	PFS
Freeman (2025) [75]	USA	-	Retrospective study	Cilta-cel/Ide-cel	RRMM	NR	183 (cilta-cel: 58, ide-cel: 125)	Delayed NT

Abbreviations: AEs, adverse events; ASCT, autologous stem cell transplantation; BCMA, B-cell maturation antigen; CI, confidence interval; CNP, cranial nerve palsy; CR, complete response; CRS, cytokine release syndrome; DOR, duration of response; HLH/IEC-HS, hemophagocytic lymphohistiocytosis/immune effector cell-associated hemophagocytic syndrome ; HRQoL, health-related quality of life; ICANS, immune effector cell–associated neurotoxicity syndrome; ICER, incremental cost-effectiveness ratio; ICU, intensive care unit; IMiD, immunomodulatory drug; IP, inpatient; ITT, intent-to-treat; LOS, length of stay; LOT, lines of therapy; MM, multiple myeloma; NDMM, newly diagnosed multiple myeloma; NR, not reported NRM, non-relapse mortality; NT, neurotoxicity; OP, outpatient; ORR, overall response rate; OS, overall survival; PFS, progression-free survival; PI, proteasome inhibitor; PPPM, per patient per month; PRO, patient-reported outcome; QoL, quality of life; RCT, randomized controlled trial; RRMM, relapsed/refractory multiple myeloma; sCR, stringent complete response; SOC, standard of care; SPM, secondary primary malignancy; Tx, treatment; USA, United States of America; VGPR, very good partial response.* Two patients received cilta-cel in the OP setting, but their results were not reported separately (one patient safety data was available).

Table S6. Patient characteristics.

Study	Trial name/ID	Treatment	Setting (IP/OP/both)	Sample size, N	Age, median (years)	Male (%)	ECOG PS (%)	Number of prior lines	Prior transplant therapy, (%)	Refractory patients, (%)
Mi (2022), Mi (2023) [42]	CARTIFAN-1/ NCT03758417	Cilta-cel	Inpatient	48	61 (30-72)	66.7	0: 45.8<br>1: 54.2	4 (3-9)	ASCT: 35.4	19
Xu (2024), Zhao (2022) [43]	LEGEND-2/ NCT03090659	Cilta-cel	Inpatient	74	54.5 (27-74)	61	0: 40.5<br>1: 43.2<br>2: 16.2	3 (1-9)	ASCT: 24.3	NR
Berdeja (2021) [8]	CARTITUDE-1 (Phase Ib/II)/ NCT03548207	Cilta-cel	Inpatient	97	61 (56-68)	59	0: 40, 1: 56, 2: 4	6 (4-8)	Auto SCT: 90<br>Allo SCT: 8	88
Martin (2022) [80]	CARTITUDE-1 (Ph II)/ NCT03548207	Cilta-cel	Inpatient	68	62 (55-70)	63	0: 40, 1: 59, 2: 2	6 (4-8)	Auto SCT: 90<br>Allo SCT: 12	88
Hillengass (2022), Hillengass (2023) [26,27]	CARTITUDE-2 Cohort A/ NCT04133636	Cilta-cel	Inpatient	20 (1 OP)	60 (38-75)	65	NR	2 (1-3)	ASCT: 85	40
Cohen (2024) [25]	CARTITUDE-2 Cohort A expansion subgroup/ NCT04133636	Cilta-cel	Inpatient	23	63 (37-74)	52.2	0: 65.2<br>1: 34.8	3 (1-3)	ASCT: 82.6	44
Van De Donk (2022), Hillengass (2023) [26]	CARTITUDE-2 Cohort B/ NCT04133636	Cilta-cel	Inpatient	19 (1 OP)	58 (44-67)	74	NR	NR	ASCT: 79	16

Study	Trial name/ID	Treatment	Setting (IP/OP/both)	Sample size, N	Age, median (years)	Male (%)	ECOG PS (%)	Number of prior lines	Prior transplant therapy, (%)	Refractory patients, (%)
Cohen (2023) [23,24]	CARTITUDE-2 Cohort C/ NCT04133636	Cilta-cel	Inpatient	20	62.5 (44-81)	60	0: 40<br>1: 60	8 (4-13)	ASCT: 100<br>Allo.SCT: 10	90
Bertrand (2024) [33]	CARTITUDE-2 Cohort D/ NCT04133636	Cilta-cel	Inpatient	17	54 (37-69)	82.4	0: 76.5<br>1: 23.5	NR	ASCT: 100	NR
San-Miguel (2023), María-Victoria Mateos (2024) [10]	CARTITUDE-4/ NCT04181827	Cilta-cel	Inpatient	176	61.5 (27-78)	55.8	0: 54.8<br>1: 44.7<br>2: 0.5	2 (1 to 3)	-	14
Schorr (2023) [59]	-	Cilta-cel	NR	9	NR	NR	NR	NR	NR	NR
Schorr (2023) [59]	-	Ide-cel	NR	25	NR	NR	NR	NR	NR	NR
Mejia Saldarriaga (2023) [60]	-	Cilta-cel	NR	25	55 (49 to 66)	NR	NR	5 (4-7)	NR	NR
Mejia Saldarriaga (2023) [60]	-	Ide-cel	NR	14	54 (48 to 58)	NR	NR	4 (4-6.75)	NR	NR
Gill (2023) [52]	-	Cilta-cel	NR	18	67.7 (52.7-81.5)	66.7	NR	5 (4-12)	Auto-SCT: 100	94.4
Gill (2023) [52]	-	Ide-cel	NR	35	70.6 (50.5-81.8)	51.4	NR	5 (4-12)	Auto-SCT: 97.1	94.4
Kocoglu (2023) [53]	-	Cilta-cel	NR	12	66	NR	NR	NR	75	NR
Kocoglu (2023) [53]	-	Ide-cel	NR	11	66	NR	NR	NR	81.8	NR
Christodoulou (2023) [54]	-	Cilta-cel/ide-cel (87% cilta-cel)	NR	15	NR	NR	NR	5 (4-10)	NR	NR
Christodoulou (2023) [54]	-	Cilta-cel	NR	13	NR	NR	NR	NR	NR	NR
Christodoulou (2023) [54]	-	Ide-cel	NR	2	NR	NR	NR	NR	NR	NR
Vieira (2023) [55]	-	Cilta-cel	NR	25	NR	NR	NR	NR	NR	NR
Attar (2023) [56]	-	Cilta-cel	NR	5	64 (40-76)	NR	NR	7 (5-8)	NR	NR
Hansen (2023) [57]	-	Cilta-cel	NR	143	64 (30-79)	57	0-1: 81.1,<br>2-4: 9.8,<br>Unknown: 9.1	6 (3-18)	AutoSCT: 121 (85)	71
Sidana (2025) [13]	-	Cilta-cel	NR	236	64	57	0-1: 89<br>2-4: 11	6 (2-18)	Prior autologous SCT: 85%	Triple-refractory: 69%<br>Penta-refractory: 30%
Wesson (2024) [41]	-	Cilta-cel	Inpatient	39	61 (56 to 67)	54	0-1: 35 (90)	6 (5 to 7)	AutoSCT: 33 (85)<br>AlloSCT: 0 (0)	NR

Study	Trial name/ID	Treatment	Setting (IP/OP/both)	Sample size, N	Age, median (years)	Male (%)	ECOG PS (%)	Number of prior lines	Prior transplant therapy, (%)	Refractory patients, (%)
Wesson (2024) [41]	-	Cilta-cel	Inpatient	90	63 (58 to 70)	52	0-1: 82 (91)	6 (5 to 8)	AutoSCT: 68 (76) AlloSCT: 7 (8)	NR
Kamboj (2024) [28]	-	Cilta-cel	NR	73	Mean (SD): 65.5 (8.1)	52	0: 16 1: 71 2: 12 3: 1.5	5 (0 to 12)	79%	NR
Dima (2025) [35]	-	Cilta-cel	NR	105	63.0 (30.0 - 76.0)	55	0-1: 94 ≥ 2: 5.8	NR	Auto-SCT: 84% ; allo-SCT: 0	Refractory to Immunomodulatory agent: 87% Refractory to Proteasome Inhibitor: 87% Double-refractory: 78% Triple-refractory: 73% Penta-refractory: 27%
Merz (2025) [30]	-	Cilta-cel	NR	42	61 (24 - 84)	50	0: 14 1: 71 2: 14	6 (4 to10)	Auto-SCT: 100%; allo-SCT: 0	Triple class: 67% Penta drug: 24%
Merz (2025) [30]	-	Ide-cel	NR	162	61 (28 - 83)	65	0: 32 1: 56 2: 11 3: 1	6 (3 to 14)	Auto-SCT: 90%; allo-SCT: 7%	Triple class: 64% Penta drug: 36%
Merz (2025) [64]	-	Cilta-cel	NR	77	65 (39 -81)	68	0: 17 1: 53 2: 22 3: 8	6 (4 to 11)	Auto-SCT: 85%; allo-SCT: 4%	NR
Merz (2025) [64]	-	Ide-cel	NR	266	64 (34 -81)	62	0: 30 1: 50 2: 16 3: 4	6 (4 to 15)	Auto-SCT: 85%; allo-SCT: 5 %	NR
Fandrei (2025) [65]	-	Cilta-cel	NR	18	65 (58–70)	44	NR	6.0 (1.0 to 8.0)	NR	Tc exposed: 11% TCRRMM: 50% Penta RRMM: 39%
Fandrei (2025) [65]	-	Ide-cel	NR	34	64 (57–67)	62	NR	7.5 (4.0 to 13)	NR	Tc exposed: 26% TCRRMM: 9% Penta RRMM: 65%
Atanackovic (2025) [34]	-	Cilta-cel	NR	23	NR	54.2	NR	5.0 (5.0)	Auto-SCT: 65.2%	NR
Atanackovic (2025) [34]	-	Ide-cel	NR	16	NR	66.7	NR	5.5 (8.0)	Auto-SCT: 68.8%	NR
Richardson (2025) [67]	-	Ide-cel	NR	10	63 (49-75)	60	NR	7 (5-12)	NR	Penta-refractory: 7%
Richardson (2025) [67]	-	Cilta-cel	NR	10	63 (49-75)	60	NR	1 (0-3)	NR	Penta-refractory: 7%

Study	Trial name/ID	Treatment	Setting (IP/OP/both)	Sample size, N	Age, median (years)	Male (%)	ECOG PS (%)	Number of prior lines	Prior transplant therapy, (%)	Refractory patients, (%)
Waldschmidt (2024) [68]	-	Cilta-cel	NR	6	NR	NR	NR	NR	NR	NR
Waldschmidt (2024) [68]	-	Ide-cel	NR	17	NR	NR	NR	NR	NR	NR
Ailawadhi (2025) [69]	-	Cilta-cel	NR	47	63.9 (9.8)	NR	NR	NR	NR	NR
Ailawadhi (2025) [69]	-	Ide-cel	NR	58	63.1 (9.5)	NR	NR	NR	NR	NR
Amoozgar (2024) [70]	-	Cilta-cel	NR	64	65.3	NR	NR	6	NR	NR
Amoozgar (2024) [70]	-	Ide-cel	NR	50	72.8	NR	NR	5	NR	NR
Hosoya (2024) [38]	-	Cilta-cel	NR	28	67 (30 to 78)	50	NR	4 (3 to 15)	NR	Penta-refractory (Cilta+Ide): 48%
Hosoya (2024) [38]	-	Ide-cel	NR	31	67 (50 to 81)	51.6	NR	6 (4 to 15)	NR	Penta-refractory (Cilta+Ide): 48%
Lim (2025) [71]	-	Cilta-cel	NR	235	NR	NR	NR	NR	NR	NR
Lim (2025) [72]	-	Cilta-cel	NR	235	NR	NR	NR	NR	NR	NR
Ailawadhi (2025) [73]	-	Cilta-cel	NR	182	Mean: 63.1	53.8	NR	5 (4-10)	NR	NR
Hansen (2025) [58]	-	Cilta-cel	NR	236	64 (30 to 84)	57	0: 30<br>1: 59<br>2: 7<br>3: 3<br>4: 0.5	NR	NR	Penta refractory status: 30%
Hansen (2025) [58]	-	Ide-cel	NR	350	65 (36 to 90)	58	0: 20<br>1: 66<br>2: 12<br>3: 2<br>4: 0.3	NR	NR	Penta refractory status: 35%
Fandrei (2024) [36]	-	Cilta-cel	NR	28	NR	NR	NR	NR	NR	NR
Fandrei (2024) [36]	-	Ide-cel	NR	34	NR	NR	NR	NR	NR	NR
Li (2024) [12]	-	Cilta-cel	NR	50	63.2 (SD 7.5)	NR	NR	NR	NR	NR
Li (2024) [12]	-	Ide-cel	NR	49	71.2 (SD 9.9)	NR	NR	NR	NR	NR
Mitch (2025) [44]	-	Cilta-cel	Inpatient	32	NR	NR	NR	NR	NR	NR
Artzenroth (2025) [39]	-	Cilta-cel	NR	24	NR	NR	NR	4 (2-9)	NR	NR
Artzenroth (2025) [39]	-	Ide-cel	NR	21	NR	NR	NR	4 (2-9)	NR	NR
Gul (2024) [37]	-	Cilta-cel	NR	172	65.5	NR	NR	≥4	NR	NR
Gul (2024) [37]	-	Ide-cel	NR	439	65.6	NR	NR	≥4	NR	NR
Kumar (2024) [29]	-	Cilta-cel (92% of 52 patients received cilta-cel)	NR	48	66 (43-83)	71%	NR	4 (1-16)	NR	NR
Kumar (2024) [29]	-	Ide-cel (4% of 52 patients received Ide-cel)	NR	2	66 (43-83)	NR	NR	NR	NR	NR

Study	Trial name/ID	Treatment	Setting (IP/OP/both)	Sample size, N	Age, median (years)	Male (%)	ECOG PS (%)	Number of prior lines	Prior transplant therapy, (%)	Refractory patients, (%)
Varga (2024) [74]	-	Cilta-cel	NR	16	NR	NR	NR	NR	NR	NR
Varga (2024) [74]	-	Ide-cel	NR	26	NR	NR	NR	NR	NR	NR
Wiemers (2024) [66]	-	Cilta-cel	NR	27	NR	NR	NR	NR	NR	NR
Wiemers (2024) [66]	-	Ide-cel	NR	34	NR	NR	NR	NR	NR	NR
Freeman (2025) [75]	-	Cilta-cel	NR	58	65 (38-83)	NR	NR	NR	NR	NR
Freeman (2025) [75]	-	Ide-cel	NR	125	65 (38-83)	NR	NR	NR	NR	NR

*Median (range); **Abbreviations:** Allo-SCT, Allogeneic Stem Cell Transplant; ASCT, Autologous Stem Cell Transplant; CAR-T, Chimeric Antigen Receptor T-cell; cilta-cel, Ciltacabtagene Autoleucel; ECOG PS, Eastern Cooperative Oncology Group Performance Status; Ide-cel, Idecabtagene Vicleucel; IP, Inpatient; NR, Not Reported; OP, Outpatient; Penta RRMM, Penta-refractory Relapsed/Refractory Multiple Myeloma; SD, Standard Deviation; SoC, Standard of Care; TCRRMM, Triple-Class Refractory Relapsed/Refractory Multiple Myeloma

Table S7. Response results for cilta-cel in RRMM patients treated in IP or unspecified treatment setting.

Author year	Trial Name/ID	Study Design	Follow-up, months	N	ORR %	CR %	PR %	Other details
Mi (2023) [42]	CARTIFAN-1/NCT03758417	Phase II trial	26.4	48	87.5	79	8	Median DOR: Not reached<br>24-mo, DOR rate: 60.6 (95% CI 42.9-74.3)
Xu (2024), Zhao (2022) [43]	LEGEND-2/NCT03090659	Phase I trial	65.4	74	87.8	73	15	Median DOR (n=65): 23.26 (95% CI 13.04-36.5)<br>Median TTR: 1.02 mo, (0.4-3.5)
Martin (2023) [80]	CARTITUDE-1/NCT03548207	Phase Ib/II trial	33.4	97	98	82	15	Median DOR: NE (23.4-NE); 24-mo DOR: 73.3 (47.2-87.9)<br>Median TTR: 1 mo, (0.7-3.3)<br>Median time-Best response: 2.6 mo (0.9-17.8)
Hillengass (2023) [26]	CARTITUDE-2 Cohort A/NCT04133636	Phase II trial	29.9	20 (1 OP)	95	90	5	Median DOR: not estimable (23.4-NE); 24-mo DOR: 73.3 (47.2-87.9)<br>Median TTR: 1 mo, (0.7-3.3)
Cohen (2024) [25]	CARTITUDE-2 Cohort A expansion subgroup/ NCT04133636	Phase II trial	15.6	22	90.9	68	23	Median DOR: not reached; 12-mo, DOR (n=20): 78.8 (52.7-91.5)* determined among pts with a >=PR<br>Median TTR: 1 mo, (0.9-9.8)
Van De Donk (2022), Hillengass (2023) [26]	CARTITUDE-2 Cohort B/NCT04133636	Phase II trial	27.9	19 (1 OP)	100	90	10	Median DOR: not estimable (23.7-NE); 24-mo DOR: 70.5 (42.5-86.7)<br>Median TTR: 0.95 mos. (0.9-9.7)
Cohen (2023) [23]	CARTITUDE-2 Cohort C/NCT04133636	Phase II trial	18.0	20	60	35	25	Median DOR: 12.3 (7.2-NE)

**Author year**	**Trial Name/ID**	**Study Design**	**Follow-up, months**	**N**	**ORR %**	**CR %**	**PR %**	**Other details**
Bertrand (2024) [33]	CARTITUDE-2 Cohort D/NCT04133636	Phase II trial	22.4	17	94.1	94	0	Median DOR: not reached<br>Median TTR: 1.3 (0.9-12.5)
Mateos et al. (2024), San-Miguel (2023) [10]	CARTITUDE-4/NCT04181827	Phase III RCT	33.6	208	84.6	73	12	Median DOR: 67.4 (59.7 to 74)
Gill (2023) [52]	-	Retrospective study	NR	18	81.8	NR	36	>= VGPR: 77.8<br>Median DOR: not reached<br>Significantly better than Ide-cel, p-value: 0.01
Kocoglu (2023) [53]	-	Prospective study	NR	12	94.4	NR	17	-
Christodoulou (2023) [54]	-	Retrospective study	NR	15	83.3	NR	NR	-
Attar (2023) [56]	-	Case-series	3.1-11	5	72.7	NR	NR	-
Hansen (2023) [57]	-	Retrospective study	5.8	143	80	40	0	-
Wesson (2024) [41]	-	Retrospective study	3.0	39	80	60	20	-
Sidana (2025) [13]	-	Retrospective study	13.0	236	89.0	70	19	-
Hansen (2025) [58]	-	Retrospective study	13.0	236	Day 30: 79<br>Day 90: 85<br>Best ORR: 89	Day 30: 26<br>Day 90: 48<br>Best: 70	NR	-
Kamboj (2024) [28]	-	Retrospective study	11.7	73	88.0	60	28	-
Dima (2025) [35]	-	Retrospective study	6.0	105	Day 30: 83<br>Day 90: 89<br>Best ORR: 92	Day 30: 27<br>Day 90: 47<br>Best: 63	Day 30: 55<br>Day 90: 42<br>Best: 30	Less partial response (<PR): Day 30: 10%,<br>Day 90: 5%
Merz (2025) [30]	-	Retrospective study	8.9	42	Day 30: 93; Day 90: 97	Day 30: 47<br>Day 90: 67	Day 30: 46<br>Day 90: 30	-
Merz (2025) [64]	-	Retrospective study	9.0	77	94.0	61	NR	-
Fandrei (2025) [65]	-	Retrospective study	3.0	18	Day 30: 94	Day 30: 61	33	SD/PD, Day 30: 1 (6%)
Atanackovic (2025) [34]	-	Prospective study	NR	23	91.0	Best: 63.6<br>Day 30: 30.4<br>Day 90: 55.4	Day 30: 52.6<br>Day 90: 35.5	-
Richardson (2025) [67]	-	Retrospective study	8.8	10	100.0	70	30	SD, n (%): 3 (30%)
Waldschmidt (2024) [68]	-	Retrospective study	4.2	6	83.0	50	NR	-
Amoozgar (2024) [70]	-	Retrospective study	NR	64	86.0	NR	NR	-

Author year	Trial Name/ID	Study Design	Follow-up, months	N	ORR %	CR %	PR %	Other details
Fandrei (2024) [36]	-	Retrospective study	10.0	28	93.0	75	NR	-
Artzenroth (2025) [39]	-	Retrospective study	NR	24	100.0	NR	NR	-

Abbreviations: CR, complete response; DOR, duration of response; mo, months; N, number of patients; NE, not estimable; NR, not reported; OP, outpatient; ORR, overall response rate; OS, overall survival; PD, progressive disease; PR, partial response; RCT, randomized controlled trial; SoC: Standard of care; SD, stable disease; TTR, time to response; VGPR, very good partial response.

Table S8. Survival outcomes for cilta-cel in RRMM patients treated in IP or unspecified treatment setting.

Author year	Trial name/ID	Study design	Follow-up, months	N	Median PFS (95% CI)	PFS Rate (95% CI)	HR (95% CI), p-value	Median OS (95% CI)	OS Rate (95% CI)	HR (95% CI), p-value	Other data
Mi (2022), Mi (2023) [42]	CARTIFAN-1/NCT03758417	Phase II trial	26.4	48	Not reached	6-mo: 85.4<br>12-mo: 77 (62.3-86.5)<br>18-mo: 66.8 (49.4-79.4)<br>24-mo: 52.6 (36.5-66.4)	-	Not reached	6-mo: 85.2, 12-mo: 78.8<br>18-mo: 78.7 (64-88), 24-mo: 74.2 (58.8-84.5)	-	-
Xu (2024) [43]	LEGEND-2/NCT03090659	Phase I/II trial	65.4	74	18.04 (10.61-26.58)	6-mo: 82.1<br>12-mo: 58.6<br>18-mo: 48.9<br>24-mo: 40.2<br>36-mo: 31.0<br>5-yr: 21 (12.2-31.4)	-	55.8 (24.4-NE)	6-mo: 90.4, 12-mo: 78.4,<br>18-mo: 66.2,<br>24-mo: 63.3,<br>36-mo: 55, 5-yr: 49.1 (37.2-60)	-	-
Lin (2023), Jagannath (2025), Berdeja (2021) [8]	CARTITUDE-1/NCT03548207	Phase Ib/II trial	61.3	97	34.9 (25.2 NE)	6-mo: 86.1, 12-mo: 75.9, 24-mo: 72.3, 36-mo: 47.5, 48-mo: 42.8, 60-mo: 36.6	-	60.7 (41.9 to not estimable)	6-mo: 92.7, 12-mo: 85.5,<br>24-mo: 72.7;<br>36-mo: 62.9,<br>48-mo: 59.6,<br>60-mo: 54.1	-	-
Hillengass (2023), Hillengass (2022), Cohen (2022) [26,27]	CARTITUDE-2 Cohort A/NCT04133636	Phase II trial	29.9	20	Not estimable (12.9-NE)	6-mo: 90 (65.6-97.4)<br>12-mo: 75<br>24-mo: 75 (50-88.7)	-	Not estimable (21.9-NE)	24-mo: 75 (50-88.7)	-	12-mo. event free rate: 79
Cohen (2024) [25]	CARTITUDE-2 Cohort A expansion subgroup/NCT04133636	Phase II trial	15.6	22	Not reached (12.3-NE)	6-mo: 81.5<br>12-mo: 77.3 (53.7-89.8)<br>18-mo: 72.3<br>24-mo: 72.3	-	Not reached (17.2-NE)	6-mo: 90.9, 12-mo: 90.9 (68.3-97.6)<br>18-mo: 80.7,<br>24-mo: 80.7	-	-
Van De Donk (2022), Hillengass (2023) [26]	CARTITUDE-2 Cohort B/NCT04133636	Phase II trial	27.9	19	Not estimable (22.6-NE)	12-mo: 90<br>24-mo: 73.3 (47.2-87.9)	-	Not estimable (NE-NE)	24-mo: 84.2 (58.7-94.6)	-	12-mo. event free rate: 84

Author year	Trial name/ID	Study design	Follow-up, months	N	Median PFS (95% CI)	PFS Rate (95% CI)	HR (95% CI), p-value	Median OS (95% CI)	OS Rate (95% CI)	HR (95% CI), p-value	Other data
Cohen (2023), Cohen (2022) [23,24]	CARTITUDE-2 Cohort C/NCT04133636	Phase II trial	18	20	9.5 (1.5-13.2)	6-mo: 60.2<br>12-mo: 38.9 (16.3-61.1)	-	16 (8.3-NE)	NR	-	-
Bertrand (2024) [33]	CARTITUDE-2 Cohort D/NCT04133636	Phase II trial	17	17	Not reached	6-mo: 100<br>12, 18 and 24-mo: 93.8 (63.2-99.1)	-	Not reached	6-mo: 100, 12: 93.8<br>18-mo: 93.8, 24-mo: 93.8 (63.2-99.1)	-	-
San Miguel (2023), María-Victoria Mateos (2024) [10]	CARTITUDE-4/NCT04181827	Phase III RCT	33.6	208	Not reached	6-mo: 83<br>12-mo: 75.9<br>18-mo: 68.9<br>24-mo: 64.4<br>30-mo: 59.4<br>36-mo: 56<br>42-mo: 54.1	Vs SoC 0.29 (0.22 to 0.39); p < 0.0001	Not reached	6-mo: 90.9<br>12-mo: 84.1<br>24-mo: 79<br>30-mo: 76.4<br>36-mo: 75.3<br>42-mo: 75.3	vs SoC 0.55 (0.39 to 0.79), p = 0.0009	-
Gill (2023) [52]	-	Retrospective study	NR	18	Not reached (NR to NR)	6-mo: 92.4	NR	Not reached (NR to NR)	NR	NR	-
Vieira (2023) [55]	-	Retrospective study	NR	25	732 days	NR	-	-	-	-	-
Attar (2023) [56]	-	Case-series	3.1 to 11	5	NR	6-mo: 75.0	-	-	-	-	-
Hansen (2023) [57]	-	Retrospective study	5.8	143	Not reached	6-mo: 79.0 (72.0-86.0)	-	Not reached	6-mo: 84.0 (78.0-91.0)	-	-
Sidana (2025) [13]	-	Retrospective study	13	236	Not reached	6-mo: 81.0, 12-mo: 68.0, 18-mo: 58.0	-	Not reached	6-mo: 91.3, 12-mo: 82.0, 18-mo: 78.1	-	-
Hansen (2025) [58]	-	Retrospective study	13	236	19.2	6-mo: 78.7<br>12-mo: 62.6<br>18-mo: 60.0	Vs Ide-cel 0.48 (0.36-0.63), p: <0.001	Not reached;	6-mo: 86<br>12-mo: 79<br>16-mo: 74.8	vs Ide-cel 0.67 (0.46-0.97), 0.03	-
Kamboj (2024) [28]	-	Retrospective study	11.67	73	NR	6-mo: 91.2<br>12-mo: 80.0<br>18-mo: 63.3<br>24-mo: 58.0<br>36-mo: 57.7	-	NR	24-mo: 72	-	-
Merz (2025) [30]	-	Retrospective study	8.9	42	Not reached	6-mo: 85.19<br>10-mo: 82<br>12-mo: 82.56	Vs. ide-cel 0.21 (0.09–0.50), <0.001	Not reached	6-mo: 90<br>10-mo: 90<br>12-mo: 90	vs ide-cel 0.24 (0.07–0.81), 0.02	-
Merz (2025) [64]	-	Retrospective study	9	77	Not reached	6-mo: 76<br>10-mo: 76<br>12-mo: 76	Vs Ide-cel (matched cohort analysis) 0.48 (0.26-	NR	10-mo: 88	NR	-

**Author year**	**Trial name/ID**	**Study design**	**Follow-up, months**	**N**	**Median PFS (95% CI)**	**PFS Rate (95% CI)**	**HR (95% CI), p-value**	**Median OS (95% CI)**	**OS Rate (95% CI)**	**HR (95% CI), p-value**	**Other data**
							0.89), 0.02				
Atanackovic (2025) [34]	-	Prospective study	NR	23	18	NR	NR	-	-	-	-
Richardson (2025) [67]	-	Retrospective study	8.8	10	Not reached	6-mo: 64.8% (95% CI: 39–100%) 12-mo: 64.8% (95% CI: 39–100%)	NR	Not reached	NR	NR	-
Amoozgar (2024) [70]	-	Retrospective study	NR	64	Not reached	NR	NR	Not reached	NR	NR	-
Ailawadhi (2025) [73]	-	Retrospective study	6.8	182	-	-	-	NR	6-mo: 97 12-mo: 91	-	-
Hosoya (2024) [38]	-	Retrospective study	11.5	28	NR	NR	NR	-	-	-	Median time to progression: 15.3 (IQR: 7.73,19.6)
Fandrei (2024) [36]	-	Retrospective study	10	28	Not Reached	NR	NR	-	-	-	-
Varga (2024) [74]	-	Retrospective study	NR	16	11 (6.2 to 11.9)	6-mo: 87 12-mo: 24 18-mo: 24	NR	-	-	-	-
Gul (2024) [37]	-	Retrospective study	NR	172	-	-	-	NR	36-mo: 37.2	vs ide-cel 1.1 (0.739–1.80), 0.86	Propensity score-matched analysis found no statistically significant difference in overall survival probabilities between Ide-cel (85.3%) and Cilta-cel (86.7%) after adjusting for confounding variables (risk difference: 0.013, 95% CI: -0.098 to 0.124, p = 0.814)

Abbreviations: CI, confidence interval; HR, hazard ratio; IQR, interquartile range; mo, months; NE, not estimable; NR, not reported; OS, overall survival; PFS, progression-free survival; RCT, randomized controlled trial; SoC, standard of care; vs, versus; yr, year.

Table S9. Safety outcomes for cilta-cel in RRMM patients treated in IP or unspecified treatment setting.

Author year	Trial Name/ID	Study Design	Follow-up, months	N	CRS %	ICANS %	DNT %	Neuromuscular Toxicity %	SPM %	HLH/IEC-HS	Infections %
Mi (2022), Mi (2023) [42]	CARTIFAN-1/NCT03758417	Phase II trial	26.4	48	Any grade: 97.9<br>Grade 3/4: 35.4<br>Median onset time: 7 days (2-10)	All grade: 2.1<br>Grade 3/4: 0	0	0	NR	6.3	Any grade: 85.4<br>COVID: 0<br>Grade 3/4: 35.4<br>The median time from cilta-cel infusion-onset infections was 205 days (range, 47-379)
Xu (2024), Zhao (2022) [43]	LEGEND-2/ NCT03090659	Phase I trial	65.4	74	Any grade: 91.9<br>Grade 1: 47.3<br>Grade 2: 35.1<br>Grade 3-4: 8.1<br>Grade 5: 1.4<br>Median time-onset: 9 days (1-19)	Grade 1: 1.3	NR	NR	5.4	NR	Viral activation: 5.4 (herpes zoster: 4.0 and hepatitis B: 1.4)
Lin (2023), Martin (2023)	CARTITUDE-1/ NCT03548207	Phase Ib/II	61.3	97	Any grade: 95<br>Grade 3-4: 4<br>Grade 5: 1<br>Median time-onset: Not reported (being checked)	NR	13 (MNT/Parkinsonism: 6.2, CNP: 1, non-MNT and non-ICANS: 6)	NR	23.7	NR	9.8
Hillengass (2023), Hillengass (2022), Janssen report (2025) [26,27,76]	CARTITUDE-2 Cohort A/NCT04133636	Phase II trial	29.9	20 (1 OP)	All grade: 95<br>Grade 3/4: 10<br>Median time-onset: 7 days (5-9)	All grade: 15<br>Grade 3/4: 0	15 (parkinsonism: 0)	Facial paralysis: 5	5	NR	NR
Cohen (2024) [25]	CARTITUDE-2 Cohort A expansion subgroup/ NCT04133636	Phase II trial	15.6	23	All grade: 100<br>Grade 3/4: 0<br>Median time-onset: 8 days (5-9)	All grade: 17.4<br>Grade 3/4: 4.3<br>Median time-onset of any grade ICANS: 10 days	0	0	8.7	NR	All grade: 34.8<br>Grade 3/4: 4.3
Van De Donk (2022),	CARTITUDE-2 Cohort	Phase II trial	27.9	19 (1 OP)	All grade: 84.2<br>Grade 3/4: 5.3<br>Median time-	All grade: 5.3	21 (parkinsonism: 5.3)	5.3	All grade:10.5	NR	NR

Author year	Trial Name/ID	Study Design	Follow-up, months	N	CRS %	ICANS %	DNT %	Neuromuscular Toxicity %	SPM %	HLH/IEC-HS	Infections %
Hillengass (2023), Van De Donk (2022), Janssen report (2025) [26,76]	B/NCT04133636				onset: 8 days (5-11)	Grade 3/4: 0			Grade 3/4: 5.3		
Cohen (2023), Cohen (2022), Van De Donk (2023) [23,24]	CARTITUDE-2 Cohort C/NCT04133636	Phase II trial	18	20	All grade: 60 Median time-onset: 7.5 days (2-10)	All grade: 20 Grade 3/4: 10 Median time-onset: 9.0 days (4-13)	0	0	NR	NR	NR
Bertrand (2024) [33]	CARTITUDE-2 Cohort D/NCT04133636	Phase II trial	22.4	17	All grade: 82.4 Grade 3/4: 0 Median time-onset: 8 days (2-10)	All grade: 5.9 Grade 3/4: 0 Median time-onset: 7	35.3 (CNP: 18)	0	5.9	NR	All grade: 70.6 Grade 3/4: 29.4
San-Miguel (2023), Dhakal (2023) [10]	CARTITUDE-4/NCT04181827	Phase III RCT	15.9	176	All grade: 76.1 Grade 3/4: 1.1	NR	17 (CNP: 9.1, parkinsonism: 1)	1	4.3	NR	NR
Van De Donk (2024), San-Miguel (2023) [10]	CARTITUDE-4/NCT04181827	Phase III RCT	21.5	176	NR	NR	NR	NR	NR	NR	Any grade: 61.5 Grade 3/4: 27.4
María-Victoria Mateos (2024), San-Miguel (2023) [10]	CARTITUDE-4/NCT04181827	Phase III RCT	33.6	176	NR	NR	NR	NR	NR	NR	Any grade: 63.5 Grade 3/4: 28.4
Mejia Saldarriaga (2023) [60]	-	Retrospective study	NR	25	During administration: 88 Grade 1/2: 95.5 Grade 3/4: 4.5	During administration: 8 Grade 1/2: 100 Grade 3/4: 0	NR	NR	NR	NR	NR
Kocoglu (2023) [53]	-	Prospective study	NR	12	Grade 1/2: 100 Grade 3/4: 0	Grade 1/2: 33 Grade 3/4: 8.3	NR	NR	NR	NR	41.7

**Author year**	**Trial Name/ID**	**Study Design**	**Follow-up, months**	**N**	**CRS %**	**ICANS %**	**DNT %**	**Neuromuscular Toxicity %**	**SPM %**	**HLH/IEC-HS**	**Infections %**
Christodoulou (2023) [54]	-	Retrospective study	3	15	Any grade: 93<br>Grade 3/4: 0	Any grade: 47<br>Grade 3/4: 7	NR	NR	NR	NR	NR
Christodoulou (2023) [54]	-	Retrospective study	NR	13	NR	NR	NR	NR	NR	NR	NR
Attar (2023) [56]	-	Case-series	3.1 to 11	5	Grade 2: 20	NR	NR	NR	NR	NR	NR
Hansen (2023) [57]	-	Retrospective study	5.8	143	Any grade: 80<br>Grade 3/4: 5	Any grade: 18<br>Grade 3/4: 6	Any grade: 12<br>Other (parkinsonism: 1, bell's palsy: 6 diplopia, dysautonomia, polyneuropathy, posterior reversible encephalopathy syndrome/progressive multifocal leukoencephalopathy): 4<br>Median onset: 25 (16-146)	NR	NR	NR	NR
Wesson (2024) [41]	-	Retrospective study	3	39	Any grade: 82<br>Grade 3/4: 13	Any grade: 23<br>Grade 3/4: 13	NR	NR	NR	NR	Any grade: 33
Dima (2025) [35]	-	Retrospective study	6	105	76	13.5	13	NR	NR	3	49
Sidana (2025) [13]	-	Retrospective study	13	236	Any grade: 75<br>Grade 3/4: 5	Any grade: 14<br>Grade 3/4: 4	10 (CNP: 5, parkinsonism: 2)	2	8.5	2	47
Merz (2025) [30]	-	Retrospective study	8.9	42	81	19	5	NR	NR	NR	NR
Merz (2025) [64]	-	Retrospective study	9	77	85	25	NR	NR	NR	NR	NR
Fandrei (2025) [65]	-	Retrospective study	3	18	67	NR	NR	NR	NR	NR	NR
Wiemers (2024) [66]	-	Retrospective study	NR	27	NR	NR	NR	NR	NR	NR	NR
Atanackovic (2025) [34]	-	Retrospective study	NR	23	87	36.3	NR	NR	NR	NR	78.3
Richardson (2025) [67]	-	Retrospective study	8.8	10	60%	0	NR	NR	NR	NR	NR
Amoozgar (2024) [70]	-	Retrospective study	NR	64	67.2	12.1	NR	NR	NR	NR	NR

Author year	Trial Name/ID	Study Design	Follow-up, months	N	CRS %	ICANS %	DNT %	Neuromuscular Toxicity %	SPM %	HLH/IEC-HS	Infections %
Lim (2025) [71]	-	Retrospective study	NR	235	Any grade: 2 (3/169)	3.6 (6/169)	13 (CNP: 8, parkinsonism: 5)	5	NR	2	NR
Lim (2025) [72]	-	Retrospective study	NR	235	NR	4% (7/173)	NR	NR	NR	NR	NR
Hansen (2025) [58]	-	Retrospective study	13	236	Any grade: 75<br>Grade 3/4: 5	Any grade: 14<br>Grade 3/4: 4	10	NR	9	NR	47
Fandrei (2024) [36]	-	Retrospective study	10	28	52	15	NR	NR	NR	NR	NR
Gul (2024) [37]	-	Retrospective study	NR	172	32.0	19.0	NR	NR	NR	NR	30.8
Kumar (2024) [29]	-	Retrospective study	NR	48	Any grade: 81<br>Grade 1: 68<br>Grade 2: 14<br>Grade 3-4: 0	Grade 1: 11<br>Grade 2-4: 0	NR	NR	NR	NR	NR
Hosoya (2024) [38]	-	Retrospective study	NR	28	85.7	28.5	NR	NR	NR	NR	NR
Freeman (2025) [75]	-	Retrospective study	15.4	58	NR	NR	5.10	NR	NR	NR	NR

Abbreviations: AE, Adverse Event; CAR-T, Chimeric Antigen Receptor T-cell; CNP: Cranial nerve palsy; CRS, Cytokine Release Syndrome; DNT, Delayed Neurotoxicity; HLH/IEC-HS: hemophagocytic lymphohistiocytosis/immune effector cell-associated hemophagocytic syndrome; ICANS, Immune Effector Cell–Associated Neurotoxicity Syndrome; IQR, Interquartile Range; mo, Months; NR, Not Reported; NT: Neurotoxicity; OP, Outpatient; RCT, Randomized Controlled Trial; SPM, Secondary Primary Malignancy.

Table S10. HCRU outcomes for cilta-cel in RRMM patients treated in IP or unspecified treatment setting.

Study details	Study design	Follow-up period	CAR-T administration setting	N	OP Visits, n (%)	Hospitalization rate, n (%)	Time-hospitalization	LOS, median	ICU admissions (%)	Reason for hospitalizations
Wesson (2024) [41]	Retrospective study	30 days	Inpatient	39	NR	8 (20.5)	NR	NR	NR	NR
Merz (2025) [30]	Retrospective study	8.9 months (6.6–11.3)	NR	42	NR	NR	NR	17 days (range, 7-69 days)	NR	NR
Atanackovic (2025) [34]	Prospective study	NR	NR	23	NR	NR	NR	NR	21.7	NR
Richardson (2025) [67]	Retrospective study	8.8 months	NR	10	NR	NR	NR	11 days (range, 0-16 days)	NR	NR
Ailawadhi (2025) [69]	Economic study	NR	NR	47	NR	NR	NR	3.9 days (SD: 4.3)	NR	NR
Sidana (2025) [13]	Retrospective study	13 months (0.3 to 21.8)	NR	236	NR	NR	NR	13 days (0-69) (Median)	8	NR

Abbreviations: AE, adverse event; CAR-T, chimeric antigen receptor T-cell; FU, follow-up; ICU, intensive care unit; IQR, interquartile range; IP, Inpatient; LOS, length of stay; MM, multiple myeloma; NA, not applicable; NR, not reported; OP, outpatient; SD, standard deviation.

Table S11. Cost outcomes for cilta-cel in RRMM patients treated in IP or unspecified treatment setting.

Study details	Study design	Country	CAR-T administration setting	Type of analysis	Cost year	Total costs	IP costs	Pharmacy costs	Office visit costs	Monitoring costs (lab and diagnostics, adverse event management costs etc.)	Other costs
Yamamoto (2024) [61]	Economic study	USA	NR	Cost-effectiveness analysis	2022 USD	1. $541,718 (Time horizon 5 years; 5-yr PFS 50%), 2. $551,614 (Time horizon 5 years; 5-yr PFS 40%), 3. $561,160 (Time horizon 5 years; 5-yr PFS 30%), 4. $572,616 (Time horizon 5 years; 5-yr PFS 20%), 5. $559,330 (Time horizon 10 years; 5-yr PFS 50%), 6. $584,534 (Time horizon 10 years; 5-yr PFS 40%), 7. $600,727 (Time horizon 10 years; 5-yr PFS 30%), 8. $612,295 (Time horizon 10 years; 5-yr PFS 20%)	NR	NR	NR	Supportive care (total): 48,212	NR
Kapinos (2023) [62]	Economic study	USA	NR	Cost-effectiveness analysis	2020 USD	$ 477,900	NR	NR	NR	NR	NR
Hansen (2024) [47]	Economic study	USA	NR	Cost per responder analysis	2023 USD	Cost per treatment patient: $704,602; Cost per responder: $963,888	NR	NR	NR	NR	559,954
Wu (2023) [51]	Economic study	China	NR	Cost-effectiveness analysis	2021 USD	$ 196,318	560	6782	115	2883	5942
Ailawadhi (2025) [69]	Economic study	USA	NR	Retrospective study	NR	$132,051 (SD:124,021) (PPPM)	NR	NR	NR	Medical costs (PPPM), mean (SD): $130,857 (124,116) Ambulatory visit costs (PPPM): $29,604 (53,381) CAR-T–related costs: 88.7% of all-cause costs	NR

**Study details**	**Study design**	**Country**	**CAR-T administration setting**	**Type of analysis**	**Cost year**	**Total costs**	**IP costs**	**Pharmacy costs**	**Office visit costs**	**Monitoring costs (lab and diagnostics, adverse event management costs etc.)**	**Other costs**
										Plan-paid costs: 99.8% of total costs	
Agostinho (2025) [63]	Economic study	Brazil	NR	Cost per responder analysis	NR	NR	NR	NR	Cost of medical resources Entry in the ER: R$ 92,30 Medical consultation: R$ 98,14 Consultation with other professionals; R$ 38,17 ICU daily cost; R$ 2.230,00	Monitoring: R$ 3.409,73	Administration costs Apheresis: R$ 12.681,06 Triage: R$ 5.432,50 Bridge therapy: R$ 283.466,20 Cryopreservation: R$ 6.376,89 Pre-medication: R$ 13.529,59 Infusion: R$ 47.291,89<br><br>Acquisition cost: R$ 2.813.351,76

Abbreviations: AE, adverse event; CAR-T, chimeric antigen receptor T-cell; CPM, cost per month; CR, complete response; ER, emergency room; FU, follow-up; ICU, intensive care unit; IP, inpatient; NR, not reported; OP, outpatient; PFS, progression-free survival; PPPM, per patient per month; pt, patient; SD, standard deviation; USD, United States dollar.

Table S12. Summary of databases and online platforms searched.

**Name of database or online platform**	**Database/online platform URL**
**Bibliographic Databases**	
MEDLINE (via Ovid)	https://ovidsp.ovid.com/
EMBASE (via Ovid)	https://ovidsp.ovid.com/
Cochrane Library	https://www.cochranelibrary.com/
**Clinical trial registries**	
NIH Clinicaltrials.gov	https://www.clinicaltrials.gov/
EU Clinical trials register	https://www.clinicaltrialsregister.eu/ctr-search/search
WHO International Clinical Trials Registry Platform (ICTRP)	https://trialsearch.who.int/Default.aspx
**Conference proceedings**	
American Society of Clinical Oncology (ASCO)	https://www.asco.org/annual-meeting
European Society for Medical Oncology (ESMO)	https://oncologypro.esmo.org/education-library
Academy of Managed Care Pharmacy (AMCP) nexus	https://amcpnexus.org/
Academy of Managed Care Pharmacy (AMCP)	https://www.amcp.org/
International Society for Pharmacoeconomics and Outcomes Research (ISPOR)	https://www.ispor.org/heor-resources/presentations-database/search
The American Society for Transplantation and Cellular Therapy (ASTCT)	https://www.astct.org/
International Myeloma Society (IMS)	https://www.myelomasociety.org/
American Society of Hematology (ASH)	https://www.hematology.org/
European Hematology Association (EHA)	https://ehaweb.org/
Society of Hematologic Oncology (SOHO)	https://sohoonline.org/SOHO/SOHO/Home.aspx

**Additional sources**	
Google Scholar	https://scholar.google.com/
